# Dysregulation of the Hippo pathway enhances PD-L2 transcription to promote immune evasion in head and neck squamous cell carcinoma

**DOI:** 10.1038/s42003-026-11092-0

**Published:** 2026-10-23

**Authors:** Toshinori Ando, Kento Okamoto, Kuniaki Sato, Yusuke Goto, Hiroki Izumi, Nanako Kataoka, Yume Ueda, Yukina Kobayashi, Kai Omachi, Yuga Maeda, Hisako Furusho, Ramiro Iglesias-Bartolome, Tetsuya Yoshimoto, Tomoaki Shintani, Souichi Yanamoto, Mutsumi Miyauchi, J. Silvio Gutkind, Mikihito Kajiya

**Affiliations:** 1 Department of Oral and Maxillofacial Pathobiology, Graduate School of Biomedical and Health Sciences, Hiroshima University, Hiroshima, Japan; 2 Department of Oral Oncology, Graduate School of Biomedical and Health Sciences, Hiroshima University, Hiroshima, Japan; 3 Moores Cancer Center, University of California, San Diego, La Jolla, CA USA; 4 Department of Urology, Chiba University Graduate School of Medicine, Chiba, Japan; 5 Department of Thoracic Oncology, National Cancer Center Hospital East, Kashiwa, Japan; 6 Center of Oral Clinical Examination, Hiroshima University Hospital, Hiroshima, Japan; 7 Laboratory of Cellular and Molecular Biology, Center for Cancer Research, National Cancer Institute, National Institutes of Health, Bethesda, MD USA; 8 Department of Pharmacology, University of California, San Diego, La Jolla, CA USA

**Keywords:** Oral cancer, Growth factor signalling

## Abstract

The Hippo pathway and its downstream effectors, YAP/TAZ, exhibit aberrant regulation in head and neck squamous cell carcinoma, facilitating cancer cell growth. However, the influence of YAP/TAZ on cancer immune evasion remains poorly understood. Herein, we show that cancer patients harboring mutations regulating the Hippo pathway and YAP/TAZ hyperactivation may be sensitized to immune checkpoint inhibitor (ICI) therapy owing to genome instability and *PD-L2* expression. LATS1/2 deletion, accompanied by YAP/TAZ hyperactivation, was associated with loss of squamous differentiation and genome instability, which may contribute to elevated DNA damage. Our data suggest that YAP/TAZ may recruit TEAD, BRD4, and RNA polymerase Ⅱ to the super-enhancer and promoter regions of *PD-L1* and *PD-L2*. YAP/TAZ predominantly contribute to PD-L2 expression, which is associated with Stat3 activation, partially enhancing *PD-L1* transcription. YAP/TAZ-activated cells can be targeted by antigen-specific T cells but evade them through PD-L2. These findings suggest that Hippo pathway alterations and associated YAP/TAZ hyperactivation may serve as potential prognostic factors for stratifying patients undergoing ICI therapy. Targeting YAP/TAZ/TEAD could offer a promising treatment avenue for cancers harboring YAP/TAZ hyperactivation.

**Figure:**
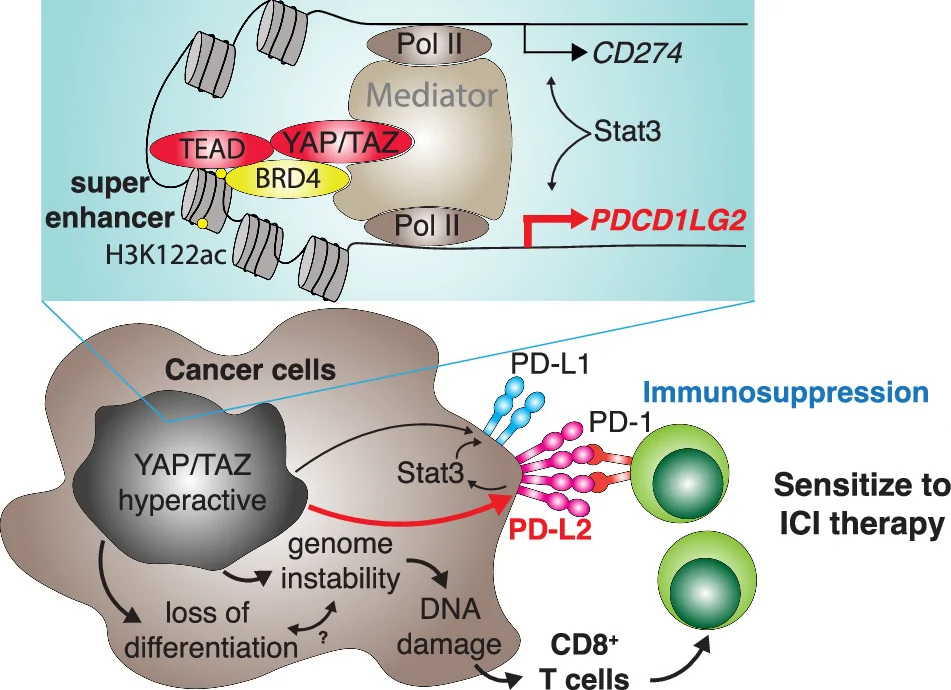

## Abstract (web summary)

We investigate how YAP/TAZ hyperactivation affects immune evasion in head and neck cancer. YAP/TAZ upregulates PD-L2 to evade T cells, highlighting these pathway alterations as potential biomarkers for ICI therapy.

## Introduction

The Hippo pathway and its downstream effectors, yes-associated protein and transcriptional coactivator with PDZ binding motif (YAP/TAZ), are indispensable for cell growth and tissue and organ development^1^. The Hippo pathway is composed of mammalian STE20-like kinases 1 and 2, large tumor suppressors 1 and 2 (LATS1/2), along with their adaptor proteins: salvador homolog 1 and MOB kinase activators 1A and 1B (MOB1A/B)^2^. The active Hippo pathway phosphorylates multiple serine residues in YAP/TAZ, leading to its cytoplasmic retention or degradation^3^. When the Hippo pathway is inactivated, dephosphorylated YAP/TAZ translocates to the nucleus, binds to TEA domain family members (TEAD), and enhances the transcription of growth-related genes, including *connective tissue growth factor* (*CTGF*) and *cysteine-rich angiogenic inducer 61* (*CYR61*)^2^. The Hippo pathway is aberrantly regulated in multiple types of cancers, resulting in YAP/TAZ hyperactivation, which initiates and promotes cancer progression^4^. For example, *YAP1* gene amplification (4.4%), *FAT atypical cadherin 1* (*FAT1*) loss or mutation (29.8%), and *Epidermal growth factor receptor* (*EGFR*) amplification (15.0%) hyperactivate YAP/TAZ in head and neck squamous cell carcinoma (HNSCC), an aggressive cancer type diagnosed in 54,540 patients that resulted in 11,580 deaths in 2023 in the United States alone^5–8^.

In addition to activation of growth-promoting pathways, cancers deploy multiple mechanisms to evade immune detection. Among them, programmed cell death 1 ligand 1 (PD-L1) in cancer cells interacts with programmed cell death 1 (PD-1) in cytotoxic T cells, thereby resulting in T cell dysfunction^9^. Immune checkpoint inhibitors (ICI) have emerged as a novel therapeutic option for many cancer types, including HNSCC^10–13^. The tumor mutational burden (TMB), which is increased by genome instability, is positively correlated with improved survival in patients receiving ICI for different cancer types^14^. PD-L1 protein levels and TMB are clinically used as predictive biomarkers; however, the response rate to ICI monotherapy remains low in HNSCC patients (less than 20%)^10,13^. Therefore, other biomarkers and targets may be required to improve the response to ICI therapy. For example, anti-PD-1 antibodies show good response rates in several cancer types, including non-small cell lung carcinoma (NSCLC) and renal cell carcinoma (RCC) regardless of PD-L1 expression^15,16^. PD-L2 has attracted attention as a novel biomarker, as it has a 2-to-6-fold higher affinity for PD-1 than that of PD-L1^17^, thereby predominantly binding to PD-1 and inducing PD-1 microcluster formation that results in T cell inactivation^18^. PD-L2 is expressed in antigen-presenting cells and cancer cells. PD-L2 is expressed at higher levels than PD-L1 in HNSCC, NSCLC, RCC, pancreatic ductal adenocarcinoma, and cervical cancer^19–23^. In particular, PD-L2 positivity is high in HNSCC, accounting for approximately 60%, almost twice as high as that of PD-L1^24^. PD-L2 levels are positively correlated with better prognosis in HNSCC patients treated with pembrolizumab, an anti-PD-1 antibody^19^. Although substantial evidence has emerged showing the importance of PD-L2 in cancer, the detailed regulatory mechanism of PD-L2 in cancer cells remains unclear.

Herein, we show that YAP/TAZ hyperactivation may be associated with genomic instability and *PD-L2* expression, which makes cancer cells susceptible to cytotoxic T cells but renders them inactive through PD-L2/PD-1 interaction, resulting in the sensitization of cancer cells to ICI therapy. Our findings suggest that Hippo pathway alterations and associated YAP/TAZ hyperactivation may serve as potential prognostic factors for stratifying patients undergoing ICI therapy. In addition, YAP/TAZ/TEAD-targeted therapy may be an effective approach for inhibiting cell growth and immune evasion.

## Results

### Alterations in genes regulating the Hippo pathway are associated with improved outcomes following immune checkpoint inhibitor therapy

**Fig. 1:**
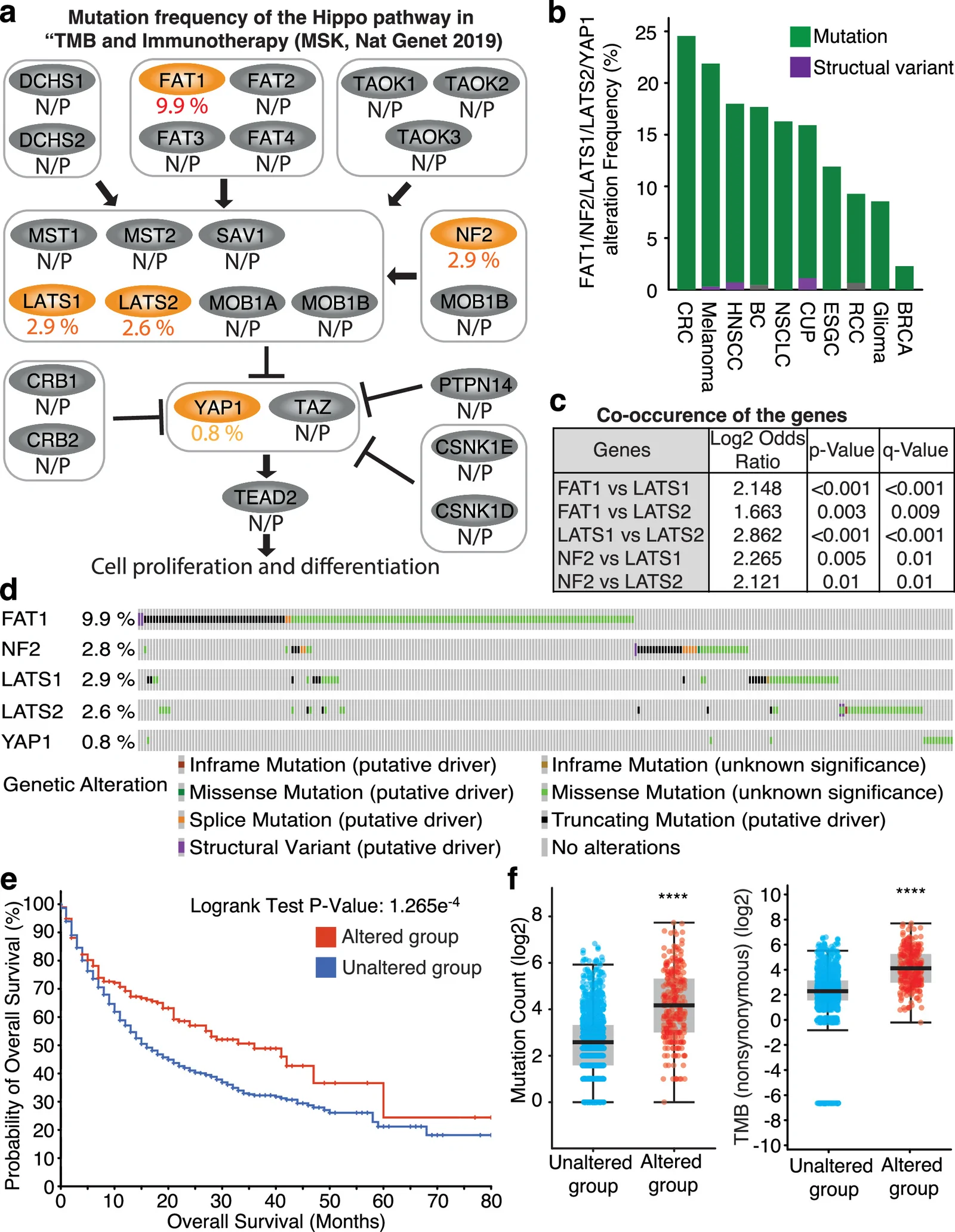
Alterations in genes regulating the Hippo pathway are associated with improved outcomes following immune checkpoint inhibitor therapy. **a** Schematic representation of mutation frequency of the genes regulating the Hippo pathway in the dataset. **b** The graph of alteration frequency of *FAT1*, *NF2*, *LATS1*, *LATS2*, and *YAP1* in various types of cancers. CRC: colorectal cancer, HNSCC: head and neck squamous cell carcinoma, BC: bladder cancer, NSCLC: non-small cell lung carcinoma, CUP: cancer of unknown primary, ESGC: esophagogastric cancer, RCC: renal cell carcinoma, BRCA: breast cancer. (**c**) Co-occurrence of the gene mutations. **d** Frequency of gene alterations of *FAT1*, *NF2*, *LATS1*, *LATS2*, and *YAP1*. **e** Overall survival of the altered group and unaltered group. The altered group contains the cancers harboring mutations of *FAT1*, *NF2*, *LATS1*, *LATS2*, and *YAP1*. **f** Mutation counts and tumor mutational burden (TMB) in the altered group (*n* = 261) and the unaltered group (*n* = 1400). Student’s *t*-test (two-sided) was used. Mean ± SEM (**f**); **** *P* < 0.0001.

First, we explored the effect of YAP/TAZ activation on cancer immune evasion using clinical and genomic data from 1,662 advanced cancer patients treated with ICI^14^. Frequently mutated components of the Hippo pathway such as *FAT1* (9.9%), *NF2* (2.9%), *LATS1* (2.9%), *LATS2* (2.6%), and *YAP1* (0.8%) were available in the data set, and analyzed (Fig. 1a). Mutations have been found in various cancer types, including colorectal cancer (CRC), melanoma, and HNSCC (Fig. 1b). Mutations in *FAT1*, *NF2*, *LATS1*, and *LATS2* co-occurred in the dataset but not in *YAP1* (Fig. 1c, d). Importantly, the altered group harboring mutations in *FAT1*, *NF2*, *LATS1*, *LATS2*, or *YAP1* exhibited a better response than the unaltered group (Fig. 1e). Interestingly, the altered group showed significantly higher mutation counts and TMB than those of the unaltered group (Fig. 1f). Following our prior clarification that EGFR activates YAP/TAZ^5^, we also examined EGFR within the same dataset. However, EGFR showed no significant differences in response to ICI therapy, mutation counts, or TMB (Supplementary Figs. 1a–c). This may be attributed to the fact that most mutations in EGFR occurred in lung adenocarcinoma (LUAC) or glioblastoma, but lacking data on EGFR amplification or overexpression, both frequently observed in HNSCC and CRC^14^. Building on the previous study’s revelation linking high TMB with improved response to ICI therapy^14^, our data suggest that YAP/TAZ hyperactivation may be associated with genome instability and elevated TMB, potentially improving the response to ICI therapy.

### LATS1/2 deletion, accompanied by YAP/TAZ hyperactivation, is associated with loss of squamous differentiation and genome instability

**Fig. 2:**
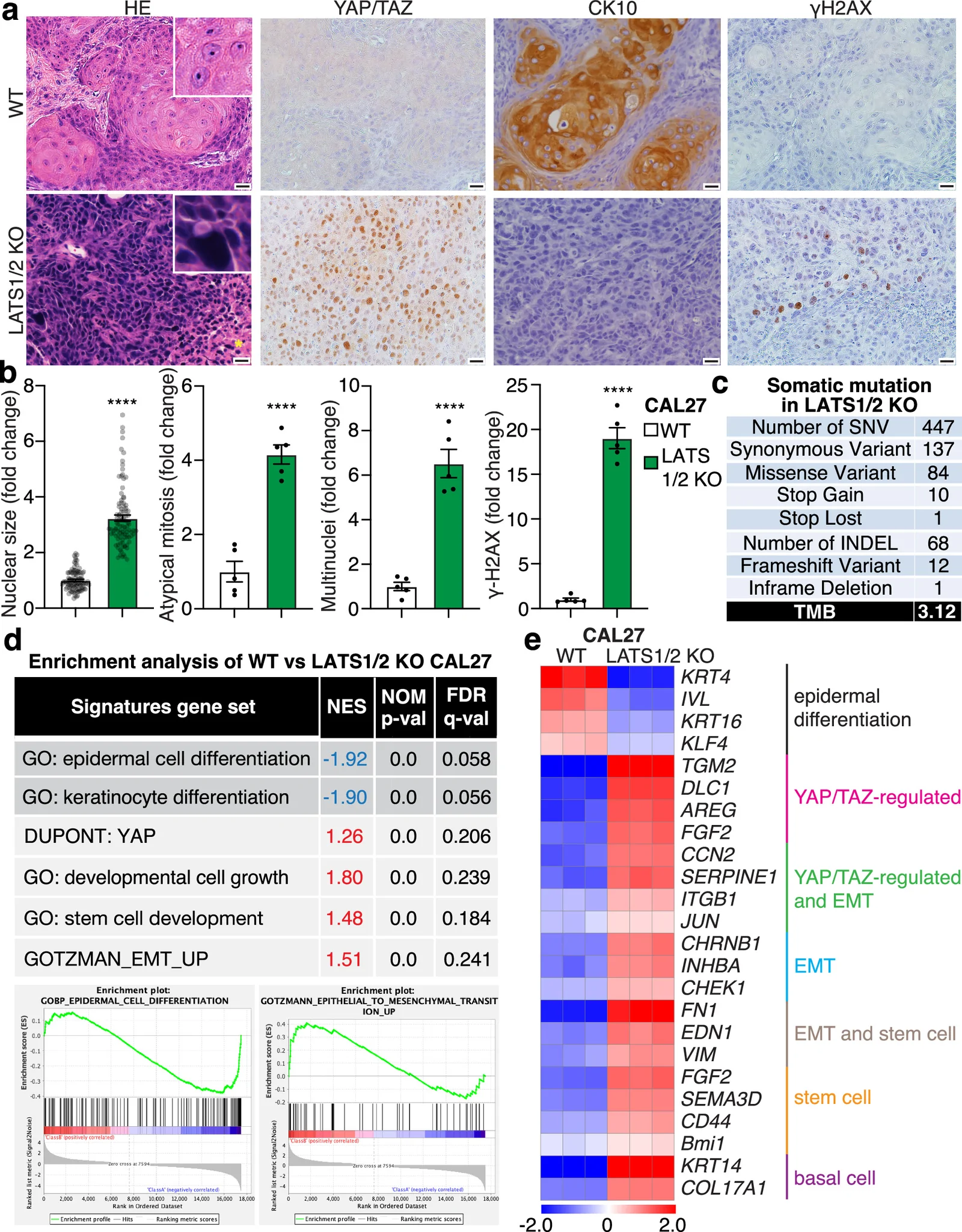
YAP/TAZ hyperactivation is associated with loss of squamous differentiation and genome instability. **a** WT and LATS1/2 KO CAL27 cells were injected into the flank of female nude mice, resulting in tumor formation, which were analyzed via HE staining and immunohistochemistry. Representative images of HE staining and immunohistochemical analysis of YAP/TAZ, CK10, and γH2AX. The yellow asterisk indicates the necrotic area. Scale bars: 20 μm. **b** The graphs depict nuclear size (*n* = 100), atypical mitosis (*n* = 5), multinuclei (*n* = 5), and positivity of γH2AX (*n* = 5) in the tissue section. **c** Somatic mutations and TMB of WT and LATS1/2 KO CAL27 cells were analyzed by conducting exome-seq. Panel displaying the comparison between LATS1/2 KO and WT results. **d** GSEA result of WT vs. LATS1/2 KO CAL27 cells (*n* = 3, statistical test was one-sided). **e** Heatmap of the representative genes of epidermal differentiation, YAP/TAZ-regulated, EMT, stem cell, and basal cell. Student’s t-test (two-sided) was used. Mean ± SEM (**b**); ****P < 0.0001.

To examine whether YAP/TAZ hyperactivation is associated with genome instability and elevated TMB, we used LATS1/2 knockout (KO) CAL27, a human HNSCC cell line that we previously generated^5^. We implanted WT and LATS1/2 KO CAL27 cells into nude mice and histologically analyzed the tumors in each group. In hematoxylin-eosin (HE) staining, the WT group displayed proliferation of well-differentiated SCC cells. Conversely, the LATS1/2 KO group exhibited proliferation of immature cells with foci of necrosis, loss of differentiation, and marked cellular atypia, including increased nuclear size, atypical mitoses, and multinuclei (Fig. 2a, b, and Supplementary Figs. 2a, b). Compared to that in the WT group, YAP/TAZ staining was more intense and accumulated in the nucleus in the LATS1/2 KO group (Fig. 2a). Furthermore, while cellular atypia was remarkably increased in LATS1/2 KO HCC827 (LUAC) cells, no distinct difference was observed in tumor differentiation. We attribute this lack of observable differentiation to the intrinsic nature of HCC827 cells, which predominantly show a solid growth pattern and intrinsically have a poor capacity to form ductal structures—a hallmark of adenocarcinoma differentiation—in in vivo xenograft models (Supplementary Fig. 2c and d). Based on these results, we hypothesized that LATS1/2 KO cells might harbor genome instability, resulting in a high TMB. Several reports have shown that γH2AX, a marker of double-stranded DNA breaks, can be a marker of genome instability and positively correlates with TMB^25,26^. Remarkably, γH2AX staining was strongly positive in the LATS1/2 KO group but almost negative in WT tumors (Fig. 2a, b). To explore the relationship between YAP/TAZ hyperactivation and TMB, we performed exome sequencing of tumors extracted from the in vivo xenograft model. Exome-sequencing revealed that somatic mutations and TMB were increased in the LATS1/2 KO group compared to those in the WT group (Fig. 2c). Gene Ontology (GO) enrichment analysis of single-nucleotide variant (SNV)/insertion-deletion (INDEL) genes in LATS1/2 KO CAL27 cells revealed enrichment in GO terms related to the regulation of cellular response to stress, including DNA repair or chromosome organization, as well as chromatin remodeling, which includes DNA damage response (Supplementary Fig. 3a). These processes are implicated in genome instability^27^.

Moreover, the WT group showed well-differentiated SCC with CK10 positivity, a marker of squamous differentiation, whereas the LATS1/2 KO group exhibited poorly differentiated or undifferentiated SCC that was almost negative for CK10 (Fig. 2a). We also performed RNA-sequencing (RNA-seq) on WT and LATS1/2 KO CAL27 cells. Gene Set Enrichment Analysis (GSEA) showed negative enrichment in GO terms related to squamous differentiation, including epidermal cell differentiation and keratinocyte differentiation, with downregulation of representative genes such as *KRT4*, *IVL*, *KRT16*, and *krüppel-like factor 4* (*KLF4*). GSEA also showed enrichment of gene sets associated with YAP-regulated (DUPONT: YAP) cell growth (GO: developmental cell growth), stem cell features (GO: stem cell development), and epithelial-mesenchymal transition (EMT) (GOTZMAN_EMT_UP), with upregulation of representative genes (Fig. 2d, e, and Supplementary Fig. 3b). GO analysis of the RNA-seq data revealed that downregulated genes in LATS1/2 KO CAL27 cells were enriched in GO terms related to epidermal differentiation, including others specific for squamous differentiation. Contrastingly, upregulated genes were enriched in GO terms associated with stem cell differentiation or development and regulation of growth (Supplementary Fig. 3c). LATS1/2 KO cells also showed increased expression of genes specific to basal cells in the epidermis, including *KRT14* and *COL17A1*^28^.

Collectively, LATS1/2 deletion, accompanied by YAP/TAZ hyperactivation, was associated with loss of squamous differentiation and genome instability in HNSCC cells.

### YAP and TAZ are predominantly associated with the expression of PD-L2 and PD-L1, respectively

**Fig. 3:**
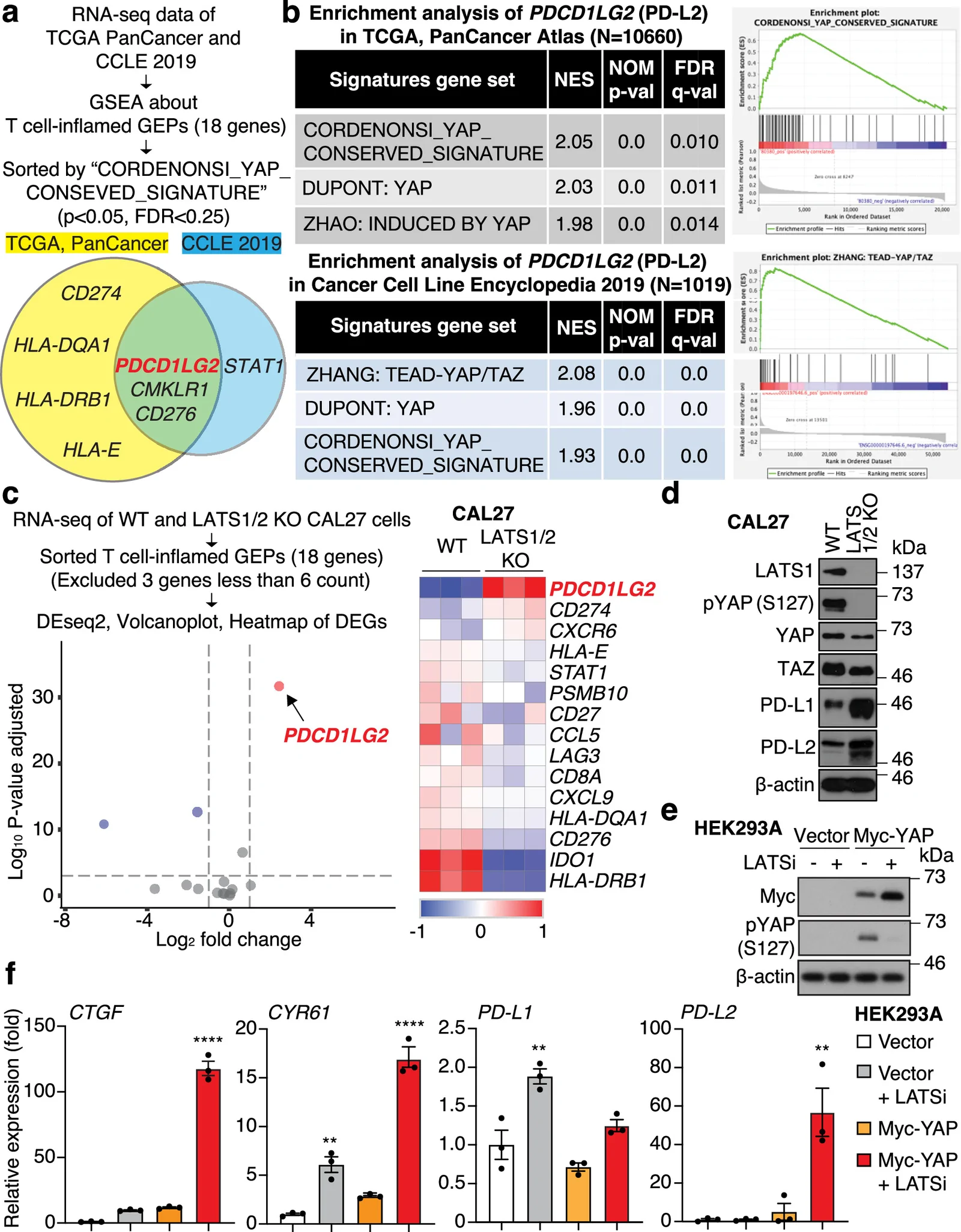
YAP and TAZ are predominantly associated with PD-L2 and PD-L1, respectively. **a** Flow of GSEA using RNA-seq data from TCGA and CCLE. **b** GSEA results showing the enrichment of YAP-regulated signature gene sets by *PDCD1LG2* in TCGA (*n* = 10660) and CCLE 2019 (*n* = 1019) (statistical test was one-sided). **c** Volcano plot and heatmap of GEPs in RNA-seq data of WT and LATS1/2 KO CAL27 cells. **d** Immunoblot of LATS1, pYAP (S127), YAP, TAZ, PD-L1, and PD-L2 in WT and LATS1/2 KO CAL27 cells. **e** Immunoblot of Myc and pYAP (S127) in HEK293A cells. Cells were transfected with vector alone or Myc-YAP. The cells were then serum-starved with treatment of LATS inhibitor (LATS-IN-1) at 10 μM for 24 h. **f** Relative mRNA expression of *CTGF*, *CYR61*, *PD-L1*, and *PD-L2*. ANOVA with Tukey−Kramer post hoc test was used (*n* = 3). Mean ± SEM (**f**); **** *P* < 0.0001; ** *P* < 0.01; *versus vector alone (**f**).

Next, we sought to clarify which other factors may contribute to the increased susceptibility of YAP/TAZ hyperactive cancers to ICI therapy. Using RNA-seq data from the Cancer Genome Atlas (TCGA), PanCancer Atlas^29^ and Cancer Cell Line Encyclopedia (CCLE) in 2019^30^, we screened T cell-inflamed gene expression profiles (GEPs) containing IFN-γ-responsive genes^31^ by performing GSEA of the YAP-regulated signature gene set (CORDENONSI_YAP_CONSERVED_SIGNATURE). *CD274* (or *PD-L1*), *HLA-DQA1*, *HLA-DRB1*, *HLA-E*, *PDCD1LG2* (or *PD-L2*), *CMKLR1*, and *CD276* were enriched in the YAP-regulated gene sets of TCGA and the PanCancer Atlas, whereas *Stat1*, *PDCD1LG2*, *CMKLR1*, and *CD276* were enriched in CCLE. *PDCD1LG2* was the most significantly and consistently enriched gene in both TCGA and CCLE (Fig. 3a and Supplementary Fig. 4a, b). *PDCD1LG2* was also significantly enriched in other YAP-regulated signature gene sets, including DUPONT: YAP, ZHAO: induced by YAP, and ZHANG: TEAD-YAP/TAZ (Fig. 3b and Supplementary Fig. 5a). To validate the correlation between *PDCD1LG2* and YAP/TAZ, we examined RNA-seq data of WT and LATS1/2 KO CAL27 cells. Among the T cell-inflamed GEPs, *PDCD1LG2* was the most highly expressed in LATS1/2 KO cells compared to WT CAL27 cells, as well as *CTGF*, *CYR61*, and *CD274* (*PD-L1*) (Fig. 3c and Supplementary Fig. 5b). The PD-L2 protein level was also higher in the LATS1/2 KO cells than in the WT CAL27 cells, as well as PD-L1 (Fig. 3d). In addition, we utilized HEK293A, a human embryonic kidney cell, to investigate direct YAP activation in the absence of activation of other cancer-associated signaling pathways. We overexpressed Myc-YAP and treated cells with LATS-IN-1 (LATSi), a LATS1/2 inhibitor, to dephosphorylate and activate YAP (Fig. 3e). The combination of Myc-YAP overexpression and LATSi treatment markedly enhanced the expression of PD-L2, CTGF, and CYR61; however, it failed to enhance PD-L1 expression (Fig. 3f). Conversely, combining HA-TAZ overexpression with LATSi treatment specifically induced *PD-L1* expression (Supplementary Fig. 6a, b), whereas *PD-L2* expression was undetectable. Collectively, these results suggest that YAP and TAZ distinctly and predominantly contribute to *PD-L2* and *PD-L1* transcription, respectively.

### YAP/TAZ/TEAD-driven PD-L2 may partially enhance PD-L1 transcription potentially through Stat3 activation

**Fig. 4:**
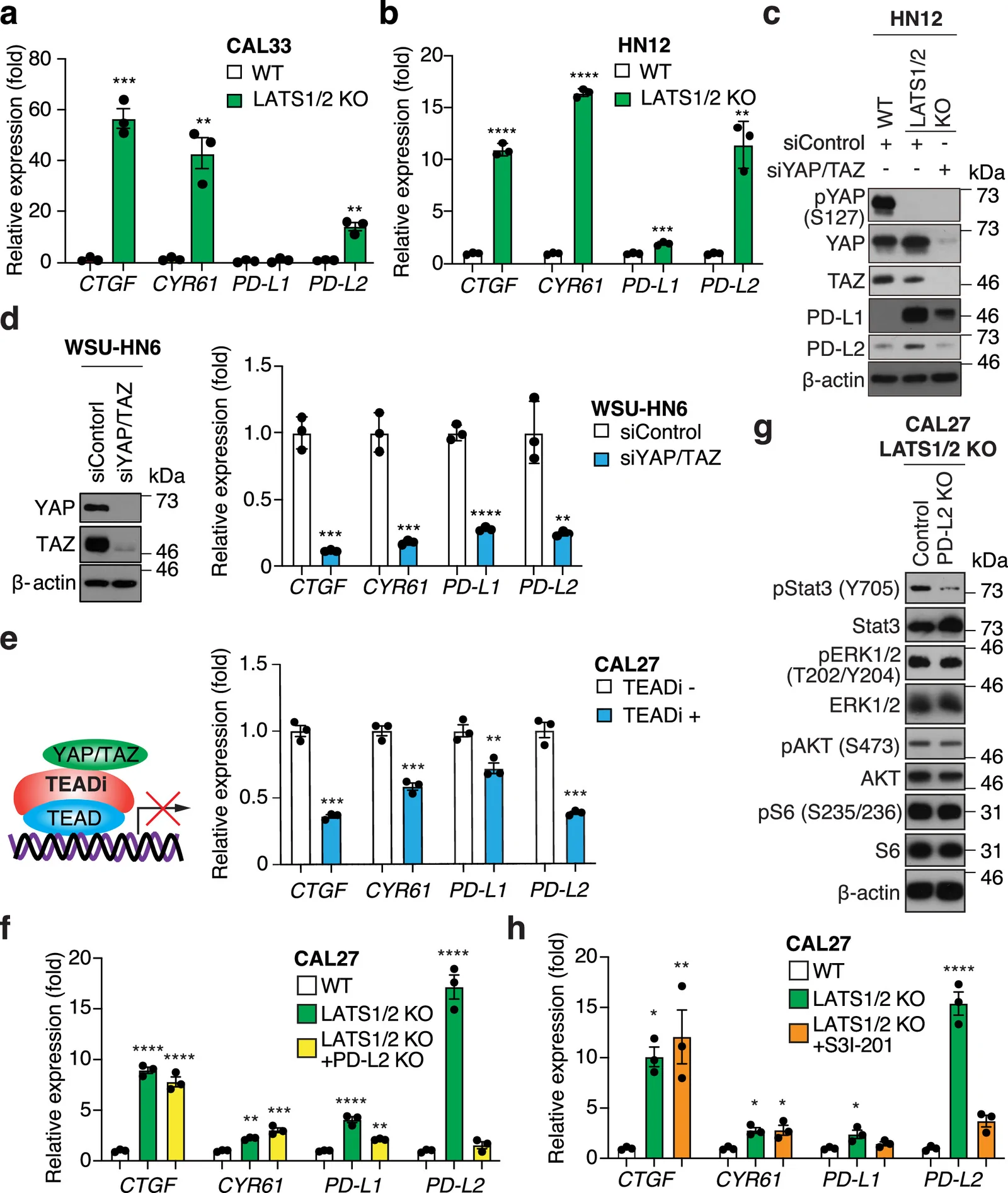
YAP/TAZ/TEAD-driven *PD-L2* partially enhances *PD-L1* transcription through Stat3 activation. **a** Relative mRNA expression of *CTGF*, *CYR61*, *PD-L1*, and *PD-L2* in WT and LATS1/2 KO CAL33 cells, and **b** HN12 cells. **c** Immunoblot of pYAP (S127), YAP, TAZ, PD-L1, PD-L2 in WT and LATS1/2 KO HN12 cells, transfected with siRNA for YAP/TAZ. **d** Immunoblot of YAP and TAZ in WSU-HN6 cells transfected with siRNA for YAP/TAZ. Relative mRNA expression of *CTGF*, *CYR61*, *PD-L1*, and *PD-L2*. **e** Schematic of TEAD inhibitor (TEADi) blocking the interaction between YAP/TAZ and TEAD. Relative mRNA expression of *CTGF*, *CYR61*, *PD-L1*, and *PD-L2* of TEADi-inducible CAL27 cells. Cells were treated with DMSO or doxycycline for 48 h. **f** Relative mRNA expression of *CTGF*, *CYR61*, *PD-L1*, and *PD-L2* in WT, LATS1/2 KO, and LATS1/2 KO + PD-L2 KO CAL27 cells. **g** Immunoblot of pStat3 (Y705), Stat3, pERK1/2 (T202/Y204), ERK1/2, pAKT (S473), AKT, pS6 (S235/236), S6, and β-actin in LATS1/2 KO and LATS1/2 KO + PD-L2 KO CAL27 cells. **h** Relative mRNA expression of *CTGF*, *CYR61*, *PD-L1*, and *PD-L2* in WT, LATS1/2 KO, and LATS1/2 KO treated with Stat3 inhibitor (S3I-201) CAL27 cells. Student’s *t*-test (*n* = 3, two-sided) was used. Mean ± SEM (**a**, **b**, **d** and **e**); **** *P* < 0.0001; *** *P* < 0.001; ** *P* < 0.01. ANOVA with Tukey−Kramer post hoc test was used (*n* = 3). Mean ± SEM (**f**, **h**); **** *P* < 0.0001; *** *P* < 0.001; ** *P* < 0.01; * *P* < 0.05; *versus WT (**f**, **h**).

To test this hypothesis, we performed further analyses using various types of cancer cells, including HNSCC, LUAC, cutaneous melanoma (CM), and RCC, all characterized by high PD-L2 expression^19^. KO of LATS1/2 or its inhibition (LATSi) consistently enhanced *PD-L2* expression in various cancer cell types, including HPV-negative HNSCC (HN12 and CAL33 cells), HPV-positive HNSCC (SCC47 cells), LUAC (HCC827 cells), CM (A375 cells), and RCC (Caki-1 cells) (Fig. 4a, b, and Supplementary Fig. 7a–d). In HN12 cells, LATS1/2 KO increased the PD-L2 protein level, which was reversed by YAP/TAZ knockdown (Fig. 4c). In contrast, the knockdown of YAP/TAZ reduced *PD-L2* expression in WSU-HN6 cells harboring *EGFR* amplification with higher YAP/TAZ activity than those of other HPV-negative HNSCC cell lines^5,32^ (Fig. 4d). We also used a doxycycline-inducible TEAD inhibitor (TEADi) possessing TEAD binding domains of VGLL4 and YAP/TAZ to disrupt the interaction between YAP/TAZ and TEAD^33,34^. Doxycycline-induced TEADi significantly suppressed *PD-L2* expression in CAL27 cells, whereas the GFP control showed no reduction (Fig. 4e and Supplementary Fig. 7e). In contrast, YAP/TAZ inconsistently regulated *PD-L1* expression. LATS1/2 KO increased mRNA and protein levels of PD-L1 in HN12 cells, LATSi treatment also increased *PD-L1* mRNA expression in A375 cells, whereas no significant changes were observed in CAL33 and Caki-1 cells, and *PD-L1* mRNA expression was instead reduced in SCC47 and HCC827 cells (Fig. 4a–c, and Supplementary Figs. 7a–d). YAP/TAZ knockdown in WSU-HN6 cells and TEADi in CAL27 cells reduced *PD-L1* expression (Fig. 4d, e). These results support the hypothesis that *PD-L1* transcription is less dependent on YAP/TAZ than *PD-L2*, which instead requires additional transcription factors to increase *PD-L1* transcript levels.

Nevertheless, *PD-L1* and *PD-L2* expression were positively correlated in TCGA, PanCancer atlas, and CCLE 2019 (Supplementary Fig. 7f). Therefore, we hypothesized that PD-L1 and PD-L2 mutually regulate their expression levels. Intriguingly, *PD-L2* KO in LATS1/2 KO CAL27 cells partially reversed *PD-L1* expression (Fig. 4f). This prompted us to examine several signaling pathways in PD-L2 KO, as *PD-L1* transcription may be regulated by many transcription factors, including MAPK, AKT-mTOR, and Stat3^35^. PD-L2 KO in LATS1/2 KO CAL27 cells showed lower phosphorylation (activation) levels of Stat3 but not of pERK, pAKT, and pS6 (Fig. 4g). PD-L2 activates Stat3 by binding to EGFR, and Stat3 accelerates *PD-L2* transcription^36,37^. Consistent with these reports, S3I-201, a Stat3 inhibitor, reversed both *PD-L1* and *PD-L2* expression in LATS1/2 KO CAL27 cells (Fig. 4h). In contrast, PD-L1 KO in LATS1/2 KO CAL27 cells did not reduce *PD-L2* expression (Supplementary Fig. 7g). Collectively, our data propose a model where YAP/TAZ predominantly upregulate *PD-L2* transcription, which is associated with Stat3 activation, potentially enhancing *PD-L1* and *PD-L2* transcription synergistically.

### YAP/TAZ/TEAD and BRD4 are implicated in PD-L1 and PD-L2 transcription through a super-enhancer

**Fig. 5:**
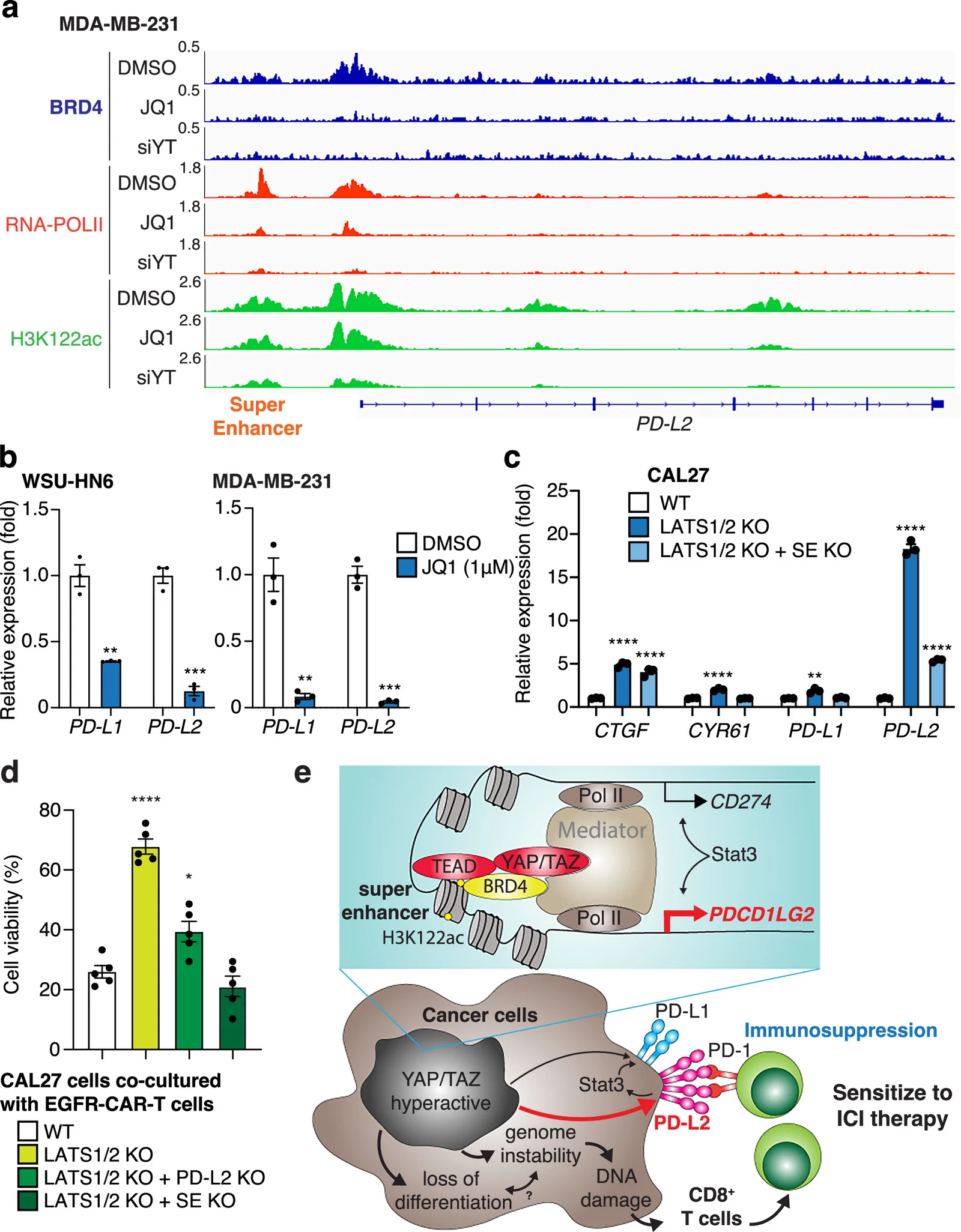
YAP/TAZ/TEAD, BRD4, and Pol Ⅱ regulate *PD-L1* and *PD-L2* transcription through the super-enhancer and promoter regions. **a** ChIP-seq result of BRD4, Pol Ⅱ, and H3K122ac in MDA-MB-231 cells. Cells were treated with DMSO, a BRD4 inhibitor (JQ1), or transfected with siRNA for YAP/TAZ. **b** Relative mRNA expression of *PD-L1* and *PD-L2* in WSU-HN6 and MDA-MB-231 cells treated with DMSO or JQ1 (1 μM) for 24 h. *n* = 3. **c** Relative mRNA expression of *CTGF*, *CYR61*, *PD-L1*, and *PD-L2* in WT, LATS1/2 KO, and LATS1/2 KO + super-enhancer (SE) KO CAL27 cells. **d** T-cell killing assay of WT, LATS1/2 KO, LATS1/2 KO + PD-L2 KO, LATS1/2 KO + SE KO CAL27 cells cultured with EGFR-CAR-T cells. **e** Schematic of YAP/TAZ-mediated immune evasion. YAP/TAZ hyperactivation drives PD-L1/L2-mediated immunosuppression, while LATS1/2 deletion induces genomic instability. Although this instability increases tumor immunogenicity, the concurrent immune evasion makes these cancer cells highly sensitive to ICI treatment. Student’s t-test (*n* = 3, two-sided) was used. Mean ± SEM (**b**); ***P < 0.001; **P < 0.01. ANOVA with Tukey−Kramer post hoc test was used (*n* = 3). Mean ± SEM (c, **d**); ****P < 0.0001; ***P < 0.001; **P < 0.01; *P < 0.05; *versus WT (**c**, **d**).

To gain mechanistic insight into *PD-L2* transcriptional regulation by YAP/TAZ, we utilized available ChIP-seq data for bromodomain-containing protein 4 (BRD4), RNA polymerase Ⅱ (Pol Ⅱ), and H3K122ac in MDA-MB-231 cells^38^. MDA-MB-231 cells harbor a homozygous mutation in neurofibromin 2 (NF2), thereby constitutively activating YAP/TAZ. MDA-MB-231 is a triple-negative breast cancer cell line, characterized by high PD-L2 levels^19^. Therefore, we used data from MDA-MB-231 cells instead of the HNSCC cell lines. Emerging evidence supports the idea that YAP/TAZ and TEAD function not only on promoters but also on enhancers. In this case, TEAD binds to super-enhancers by recruiting BRD4 and Pol Ⅱ at YAP/TAZ-regulated promoters^38^. BRD4 has acetyltransferase activity, resulting in the acetylation of K122 in histone H3 (H3K122ac)^39^, and in turn, H3K122ac induces Pol Ⅱ loading onto promoters, resulting in transcriptional activation^40^. In contrast, the super-enhancer located between *PD-L1* and *PD-L2* is responsible for transcription of *PD-L1* and *PD-L2*^41^. Given the above evidence, we hypothesized that YAP/TAZ and TEAD regulate PD-L1 and PD-L2 by controlling the super-enhancer and promoter regions. We focused on the super-enhancer located between *PD-L1* and *PD-L2* in the ChIP-seq data. As expected, BRD4 and Pol Ⅱ were enriched at the super-enhancer and promoter regions of *PD-L2*, in line with H3K122ac. JQ1, an inhibitor of BRD4, and YAP/TAZ knockdown by siRNA inhibited the recruitment of BRD4, Pol Ⅱ, and H3K122ac to the super-enhancer and promoter regions of *PD-L2* (Fig. 5a). BRD4 and Pol Ⅱ were also loaded onto the promoter region with H3K122ac of *PD-L1*, which was reduced by JQ1 treatment and YAP/TAZ knockdown; however, Pol Ⅱ was not inhibited by JQ1 treatment (Supplementary Fig. 8a). We further validated the mechanism by which YAP/TAZ and BRD4 regulate *PD-L1* and *PD-L2* expression using in vitro cell-line models. JQ1 treatment significantly suppressed *PD-L1* and *PD-L2* mRNA expression in WSU-HN6 and MDA-MB-231 cells (Fig. 5b). JQ1 treatment also reduced their mRNA and protein levels in LATS1/2 KO CAL27 cells (Supplementary Figs. 8b, c). In addition, YAP/TAZ knockdown reduced *PD-L1* and *PD-L2* mRNA and protein levels in MDA-MB-231 cells (Supplementary Figs. 8d, e). In situ proximity ligation assay (PLA) revealed that YAP and BRD4 interact in the nucleus in LATS1/2 KO CAL27 cells (Supplementary Fig. 8f). To evaluate the role of the super-enhancer between *PD-L1* and *PD-L2*, we knocked out the super-enhancer (SE KO) in LATS1/2 KO CAL27 cells. SE KO reversed the LATS1/2 KO-associated upregulation of *PD-L1* and *PD-L2* expression (Fig. 5c).

Finally, we performed a T-cell killing assay to explore the biological function of YAP/TAZ-associated *PD-L2* expression in immune evasion. WT CAL27 cells were susceptible to EGFR-CAR-T cell-mediated attack, resulting in decreased cell viability, whereas LATS1/2 KO cells escaped this effect. As we previously demonstrated, LATS1/2 deletion does not alter total EGFR expression levels in multiple cancer cell lines^5^, the observed differences in CAR-T susceptibility are attributed to PD-L1/PD-L2 upregulation rather than the loss or downregulation of the target antigen. Furthermore, PD-L2 KO in LATS1/2 KO cells increased their sensitivity to EGFR-CAR-T cells, and SE KO further reduced their viability to a level comparable to that of WT CAL27 cells (Fig. 5d). Collectively, these data suggest that even when targeted by antigen-specific T cells (such as EGFR-CAR-T cells), LATS1/2-deficient cells effectively evade this cellular immunity predominantly through PD-L2 expression.

## Discussion

We propose a model based on our findings that YAP/TAZ hyperactivation predominantly promotes *PD-L2* expression, potentially through the super-enhancer and regulation of its promoter by TEAD, BRD4, and Pol Ⅱ. Furthermore, we suggest that PD-L2 partially contributes to increased *PD-L1* expression via Stat3 activation. In addition, LATS1/2 deletion, accompanied by YAP/TAZ hyperactivation, was associated with the loss of squamous differentiation and genome instability. While genome instability and elevated DNA damage may increase the immunogenicity of cancer cells and promote CD8^+^ T cell recognition, the cancer cells can then evade immune clearance via PD-L1/PD-L2-mediated immunosuppression, thereby rendering them highly sensitive to ICI treatment (Fig. 5e).

Compared to well-differentiated SCC, poorly differentiated SCC lesions generally show central necrosis of tumor nests and proliferation of immature cells with marked nuclear pleomorphism and atypical mitoses without apparent keratinization^42^. However, the regulatory mechanism by which the loss of squamous differentiation and cytological atypia occur simultaneously remains unclear. Interestingly, our results are consistent with the general histological findings of SCC; LATS1/2 deletion, accompanied by hyperactive YAP/TAZ, was associated with both marked cellular atypia and loss of squamous differentiation, which might cooperatively contribute to genome instability (Fig. 2a–c). Emerging evidence suggests that YAP/TAZ activation inhibits KLF4, sustaining basal cell proliferation, and blocks squamous differentiation^33,34^. Consistent with these reports, LATS1/2 KO CAL27 cells reduced *KLF4* expression (Fig. 2e), suggesting it may be involved in the loss of squamous differentiation by LATS1/2 deletion associated with YAP/TAZ hyperactivation. On the other hand, several studies have shown that YAP/TAZ hyperactivation induces aneuploidy and genome instability^43–47^. SNVs/INDELs in LATS1/2 KO CAL27 cells were enriched for genes involved in DNA repair or chromatin remodeling (Supplementary Fig. 3a), which might contribute to genome instability. Further transcriptomic analysis is required to clarify the primary regulators that trigger the loss of squamous differentiation and genome instability associated with LATS1/2 deletion.

Furthermore, our current study unveils a sequential, unidirectional cascade linking YAP/TAZ activation to immune checkpoint regulation via the Stat3 pathway. While some previous reports have suggested a potential reciprocal feedback loop where Stat3 can regulate YAP/TAZ activity in certain contexts^48^, our data demonstrate a distinct mechanism in our OSCC model. Specifically, Stat3 activated downstream of the YAP/TAZ–PD-L2 axis then synergistically enhances the transcription of both PD-L1 and PD-L2. Importantly, consistent with our previous findings^32^, Stat3 inhibition does not alter YAP activation status, ruling out a positive feedback loop from Stat3 back to YAP/TAZ in this setting. Collectively, this YAP/TAZ-PD-L2-Stat3-PD-L1/PD-L2 axis highlights a novel layer of complexity in tumor immune evasion, suggesting that targeting the downstream Stat3 pathway could be a highly effective therapeutic strategy to disrupt YAP/TAZ-driven immunosuppression.

We first elucidated the mechanism by which YAP/TAZ-hyperactivated cancer cells evade antigen-specific T cell attack through the PD-L2/PD-1 interaction, even when they are highly susceptible to immune recognition. Moroishi et al. reported that LATS1/2 deficient cells release nucleic acid-rich extracellular vesicles, inducing a type Ⅰ interferon response and recruiting cytotoxic T cells to enhance anti-tumor effects^49^. In contrast to prior studies, our results showed that LATS1/2 KO cells can escape from EGFR-CAR-T cells via YAP/TAZ-associated PD-L1 and PD-L2, suggesting that YAP/TAZ hyperactivity can accelerate cancer initiation, progression, and immune evasion. This possibility is further supported by recent discoveries demonstrating that MOB1 KO mice rapidly develop SCC on the tongue, as well as by expression of active YAP (S127A) and HPV E6/E7 in transgenic mice^50,51^. However, the effect of YAP/TAZ activation on the regulation of the tumor microenvironment (TME) remains unclear in this study. Various reports suggest that YAP/TAZ-hyperactive cancer cells regulate not only cytotoxic T cells but also NK cells, macrophages, regulatory T cells, and myeloid-derived suppressor cells (MDSCs) in the TME; however, this is still not fully understood^52^. Further detailed analysis using syngeneic mouse models bearing YAP/TAZ-hyperactive cancers, including HNSCC, is required to clarify the effect of YAP/TAZ hyperactivation in cancer cells on the TME.

There are several limitations to our study. Primarily, our analysis was based on the MSKCC clinical genomic dataset, which is restricted to somatic mutation, TMB, and survival data. Consequently, we were unable to directly verify the functional activation of YAP/TAZ—such as through transcriptomic profiling or assessing subcellular localization via immunohistochemistry—in patients harboring Hippo pathway mutations. Furthermore, due to the lack of matched tissue samples, we could not directly validate the relationship among YAP/TAZ activation, PD-L1/PD-L2 expression, and immune cell infiltration in clinical settings. Future studies utilizing independent clinical cohorts with matched multi-omics and tumor microenvironment data are warranted to fully translate our in vitro findings into clinical applications.

Another important aspect of our study is the reliance on a cohort treated exclusively with ICIs (without concurrent cytotoxic chemotherapy). In current clinical practice, ICIs are increasingly combined with cytotoxic chemotherapy, making large cohorts treated solely with immunotherapeutic agents rare. However, to accurately assess pure immunological responses and the true prognostic value of genomic alterations like Hippo pathway mutations, it is essential to exclude the confounding effects introduced by concurrent chemotherapies. Therefore, despite the clinical shift towards chemo-immunotherapy combinations, we emphasize that continuing to prospectively collect and analyze cases of pure ICI regimens remains crucial for the definitive validation of such genomic prognostic factors.

Mechanistically, our data propose a model wherein YAP/TAZ predominantly facilitate PD-L2 expression through a super-enhancer region, potentially orchestrated by a transcriptional complex involving TEAD, BRD4, and Pol II. While a previous study reported that YAP physically interacts with BRD4 to drive pro-tumorigenic target genes in breast cancer^38^, our study extends this paradigm to immune evasion mechanisms. By performing an in situ proximity ligation assay (PLA), we provided direct evidence of a physical interaction between YAP and BRD4 in LATS1/2 KO CAL27 cells (Supplementary Fig. 8f). This critical finding supports a dynamic molecular model: upon YAP/TAZ binding to TEAD at the PD-L2 super-enhancer, this complex may actively recruit the epigenetic reader BRD4. BRD4, in turn, might enhance the recruitment and activation of Pol II at the PD-L2 promoter, thereby contributing to robust and sustained PD-L2 transcription.

Furthermore, elucidating this specific YAP-BRD4-PD-L2 axis reveals novel downstream implications and therapeutic vulnerabilities. Because BRD4 is essential for the super-enhancer-mediated upregulation of PD-L2, pharmacological disruption of this interaction—for instance, using BET inhibitors—could effectively dissociate the transcriptional complex. This approach may not only abrogate YAP/TAZ-driven PD-L2 expression but also suppress other downstream oncogenic programs orchestrated by YAP/TAZ. Thus, targeting the YAP-BRD4 interaction represents a promising and novel strategy against YAP/TAZ-hyperactivated HNSCC.

Based on our results, targeting PD-L1, PD-L2, or PD-1 may be a reasonable therapeutic approach for cancer patients with hyperactive YAP/TAZ, including HNSCC. Anti-PD-1 antibodies have already been approved for the treatment of various types of cancers, including HNSCC. Patient stratification based on YAP/TAZ activation may improve the selection of patients more likely to respond to anti-PD-1 immunotherapy. In addition, recent studies have elucidated the importance of PD-L2 as a therapeutic target. PD-L2, but not PD-L1, can bind to another receptor, repulsive guidance molecule B (RGMb), which is expressed on tumor-infiltrating cytotoxic CD8^+^ T cells, thereby inhibiting CD8^+^ T cell activity^53,54^. Chemotherapy-induced intratumoral senescent cells overexpress PD-L2, which accumulates MDSCs for immune evasion^55^. Given this body of evidence, targeting PD-L2 itself or its receptors, PD-1 or RGMb, in patients with YAP/TAZ-hyperactive cancers may represent a reasonable approach. A phase 2 clinical trial recently demonstrated that combining pembrolizumab, an anti-PD-1 antibody, with cetuximab, an anti-EGFR antibody, achieved a high overall response rate of 45% in patients with recurrent or metastatic HNSCC, regardless of PD-L1 expression levels^56^. Given our results that YAP/TAZ hyperactivation drives PD-L2 expression and that EGFR inhibition may potentially trigger compensatory YAP/TAZ activation, this combination therapy could be particularly effective in tumors where the YAP/TAZ-PD-L2 axis is active. Thus, targeting the YAP/TAZ pathway through dual EGFR and PD-1/PD-L2 blockade represents a rational therapeutic strategy to overcome immune evasion.

On the other hand, direct targeting of the Hippo pathway, YAP/TAZ, or TEAD has been attracting attention for drug discovery in the cancer research field. Recent studies have shown that TEAD is auto-palmitoylated at its cysteine residues, which are indispensable for binding to YAP/TAZ^57^. Therefore, TEAD inhibitors targeting auto-palmitoylation have emerged as novel drugs that disrupt protein-protein interactions between YAP/TAZ and TEAD. Our data showed that a doxycycline-inducible TEAD inhibitor suppressed the expression of *PD-L1* and *PD-L2*. Therefore, TEAD inhibitors may exhibit a dual function of anti-proliferative and anti-immune evasion, suggesting that TEAD inhibitor monotherapy functions similarly to combination therapies such as cytotoxic chemotherapy and anti-PD-1 antibodies. Further research is warranted to validate the synergistic effects of TEAD inhibitors and ICI therapeutics.

Collectively, we provide evidence of a novel mechanism by which YAP/TAZ promotes *PD-L2* expression, which is essential for immune evasion. These findings indicate that Hippo pathway alterations and the putative YAP/TAZ hyperactivation could potentially be associated with favorable responses to ICI therapy. Moreover, TEAD inhibitors with both antiproliferative and immune-evasion-inhibitory effects may be promising therapeutic approaches for patients with YAP/TAZ-hyperactive cancers, including HNSCC.

## Materials and Methods

### Antibodies and reagents

Anti-pYAP (S127) (#4911, 1:2000), YAP (#14074, 1:1000), LATS1 (#3477, 1:1000), PD-L1 (#29122, 1:500), PD-L2 (#82723, 1:500), pStat3 (#9131, 1:1000), Stat3 (#9139, 1:1000), pERK (T202/Y204, 1:1000) (#4376), ERK1/2 (#4696, 1:1000), pAKT (S473) (#4060, 1:1000), AKT (#9272, 1:1000), pS6 (S235/236) (#4696, 1:2000), S6 (#2217, 1:1000), and Myc-tag (#2276, 1:1000) were purchased from Cell Signaling Technology (MA, USA). β-actin (#A2228, 1:10000) was acquired from Sigma-Aldrich Inc. (MO). LATS-IN-1 (#HY-138489) was obtained from MedChemExpress (NJ), and JQ1 (#S9742) was purchased from Selleck Chemicals (TX, USA).

### DNA constructs and generation of stable DOX-inducible TEADi-expressing cells

The plasmid pQCXIH-Myc-YAP (Addgene33091 from Kunliang Guan’s lab)^58^ was used. pInducer20 EGFP TEADi (Addgene #14015) was a gift from Ramiro Iglesias-Bartolome^33,34^. For lentivirus production, 293 T cells were plated onto a poly-D-lysine-coated 10 cm dish 16 hours prior to transfection. Cells were co-transfected with 3 µg VSV-G (Addgene #14888), 6 µg psPAX2 (Addgene #12260), and 9 µg pInducer20 EGFP TEADi using 36 µL P3000 reagent and 43.4 µL Lipofectamine 3000 according to the manufacturers’ protocols. Culture media were refreshed 16 hours post-transfection. At 48- and 72-hour time points, virus-containing media were collected and filtered through a 0.45 µm PVDF membrane filter and stored at 4 °C. Lentivirus suspension was then concentrated using Lenti-X Concentrator (Takara Bio) according to the manufacturer’s protocols. Lentivirus was resuspended in 500 µL PBS and stored at -80°C until the time of transduction. For generation of stable cell lines, CAL27 cells were plated onto a 10 cm plate 16 hours prior to lentiviral transduction. Subsequently, 50 µL of lentivirus-containing PBS and 10 µL polybrene (10 mg/ml, Sigma) were added to the culture medium. 24 hours after transduction, the culture medium was refreshed, and cells were selected with 1000 µg/ml G418 (Invivogen) for 10 days. To express DOX-inducible TEADi, cells were treated with 1 µg/ml Doxycycline (Sigma) for 48 hours.

### CRISPR/Cas9 genome editing

LATS1/2 KO cells were created using pLentiCRISPRv2 expressing CAS9 and sgRNA as described previously^59^.

### Cell culture

CAL33, CAL27, HN12, WSU-HN6, SCC47, A375, and HEK293A cells were kindly supplied by J. Silvio Gutkind. Caki-1 and HCC827 cells were sourced from Yusuke Goto and Hiroki Izumi, respectively, with their identities confirmed via STR profiling. They were also verified as free of Mycoplasma infection. CAL33, CAL27, HN12, WSU-HN6, SCC47, A375, and HEK293A cells were cultured in Dulbecco’s modified Eagle’s medium (D-6429, Sigma-Aldrich Inc., St. Louis, MO, USA), and Caki-1 and HCC827 cells were cultured in RPMI 1640 medium (30264-56, nacalai Tesque, Kyoto, Japan) supplemented with 10% fetal bovine serum (Sigma-Aldrich Inc., St. Louis, MO) and 1x antibiotic/antimycotic solution (Sigma-Aldrich Inc., MO).

### Western blotting

Western blotting was performed as described previously^32^.

### Cell viability assay

Cell viability assay was performed as described previously^32^.

### Crystal violet staining

Crystal violet staining was performed as described previously^59^.

### RNA isolation and real-time PCR

RNA isolation and real-time PCR were performed as described previously^59^.

The following primers were used. GAPDH F: 5′-GAGTCAACGGATTTGGTCGT-3′, R: 5′-TTGATTTTGGAGGGATCTCG-3′; CTGF F: 5′-GTTTGGCCCAGACCCAACTA-3′, R: GGCTCTGCTTCTCTAGCCTG-3′; CYR61 F: 5′-CAGGACTGTGAAGATGCGGT-3′, R: GCCTGTAGAAGGGAAACGCT-3′; PD-L1 F: 5′- CAATGTGACCAGCACACTGAGAA-3′, R: GGCATAATAAGATGGCTCCCAGAA-3′; PD-L2 F: 5′- AAAGACCTGTCACCACAACAAAG-3′, R: AAAGTGCTGGGTCATCCAAAG-3′

### RNA-seq

Previously generated RNA-seq data were analyzed as previously described^59^.

### Gene Set Enrichment Analysis

For the analysis of RNA-seq data from TCGA^6^ and CCLE^30^, GSEA (Broad Institute http://software.broadinstitute.org/gsea/index.jsp) was performed using 1000 permutations, the “Pearson” metric of RNA-seq read counts per gene, and a gene set size filter of 15-500. The “C6” gene set database from MSigDB (CORDENONSI_YAP_CONSERVED_SIGNATURE) was spiked with “DUPONT: YAP”^60^, “ZHAO: Induced_by_YAP”^61^, and “ZHANG: TEAD-YAP/TAZ” (“C6 modified”). For analysis of the RNA-seq data, GSEA was performed as described above using a *t*-test metric for ranking genes. The C2, C5, and modified C6 gene-set collections were used.

### Exome-seq

Genomic DNA was extracted using NucleoSpin Tissue (Takara Bio, San Jose, CA). To generate standard exome capture libraries, the Agilent SureSelectXT Low Input Target Enrichment protocol for the Illumina paired-end sequencing library with 1 µg of input gDNA was used. DNA quantity and quality were measured using PicoGreen and agarose gel electrophoresis, respectively. We used 1 μg of each cell line’s genomic DNA diluted with EB Buffer and sheared to a target peak size of 150–200 bp with the Covaris LE220 focused-ultrasonicator (Covaris, Woburn, MA) according to the manufacturer’s recommendations. Fragmentation was followed by end-repair and the addition of an “A” tail. Agilent adapters were then ligated to the fragments, and after assessing the efficiency of ligation, the adapter-ligated product was PCR amplified. The final purified product was quantified using TapeStation DNA ScreenTape D1000 (Agilent Technologies). For exome capture, 250 ng of DNA library was mixed with hybridization buffers, blocking mixes, RNase block, and 5 µL of SureSelect all exon capture library, according to the standard Agilent SureSelect Target Enrichment protocol. Captured DNA was washed and amplified. The final purified product was then quantified via qPCR according to the qPCR Quantification Protocol Guide (KAPA Library Quantification kits for Illumina Sequencing platforms) and qualified using a TapeStation DNA ScreenTape D1000 (Agilent).

### Data analysis and sample filtering

For the DNA-seq studies, paired-end sequences produced by NovaSeq Instruments were mapped to the human reference genome (hg38 from UCSC). PCR duplicates were removed using the MarkDuplicates.jar in the “Picard-tools” package (picard-tools-2.18.2-SNAPSHOT), which requires reads to be sorted. Reads with identical starting positions were considered duplicates and reduced to a single read. The BAM files were then recalibrated using Base Quality Score Recalibration, which uses machine learning to model sequencing errors empirically and adjust the quality scores accordingly. Based on the previously generated BAM file, variant genotyping for each sample was performed using the HaplotypeCaller of GATK (GATKv4.0.5.1). At this stage, SNP and short indel candidates were detected at nucleotide resolution. Variants were filtered using VariantFiltration in the GATK Tool. Filtered variants were annotated using another program called SnpEff (SnpEff 4.3t 2017-11-24) and filtered using dbSNP (138, 151) and SNPs from the 1000 Genome project (Phase3). The format of the final product was vcf. An in-house program and SnpEff were used to annotate additional databases, including ESP6500 (ESP6500SI_V2), ClinVar (07/2018), dbNSFP (dbNSFPv3.5c), and ACMG information.

### In vivo mouse experiment

The tumor xenograft study was performed according to the protocol (#A22-29) approved by the Hiroshima University Research Facilities of Laboratory Animal Science. Female and male athymic nude mice (BALB/cAJcl-nu/nu, 5–6 weeks old) were purchased from CLEA Japan (Shizuoka, Japan). WT and LATS1/2 KO CAL27 cells were transplanted into both flanks (2 million cells per tumor) of each mouse. When the average tumor volume reached a predetermined value (~150 mm^3^), the mice were randomized into groups (five tumors per group). The mice were euthanized on day 21, and the tumors were extracted. In the HE-stained tumor tissue sections, nuclear size was measured for 100 cells. Additionally, atypical mitosis and multinuclei of the tumor cells were counted across five high-power fields (400x magnification).

### T-cell killing assay

CAR-T Cells, EGFRscFv-4-1BB-CD3z (PM-CAR1022), were obtained from ProMab Biotechnologies, Inc. (CA, USA). CAL27 cells were seeded and co-cultured with EGFR-CAR-T cells at a 1:5 ratio (cancer cells to EGFR-CAR-T cells). EGFR-CAR-T cells were washed with PBS, and the remaining cancer cells were assessed using a cell viability assay.

### Statistical analysis

GraphPad Prism version 9 for Windows (GraphPad Software, CA) was used for statistical analysis. The data were analyzed using Student’s *t*-test (two-sided) and analysis of variance (ANOVA) with Tukey–Kramer post hoc test. All experiments were repeated independently three times with similar results.

## Supplementary information

Supplementary Information

Description of Additional Supplementary Files

Supplementary Data 1

nr-reporting-summary

## Data Availability

Raw and processed RNA-seq data were deposited in the NCBI Gene Expression Omnibus (GEO) and can be accessed under the GEO Series Accession Number GSE (GSE268568). The ChIP-seq data were acquired from the GEO (GSE102409) database. The CCLE dataset is available online (https://portals.broadinstitute.org/ccle). TCGA data are available from c-bioportal (https://www.cbioportal.org/). Supplementary Materials and Methods, Supplementary Figs. 1–8, and uncropped western blot images are provided in the Supplementary Information. Source data for the figures in this paper are provided in Supplementary Data 1. All data supporting the findings of this study are available from the corresponding author upon reasonable request.
