# Supplementary material for: Dysregulation of the Hippo pathway enhances PD-L2 transcription to promote immune evasion in head and neck squamous cell carcinoma
Source: Commun Biol. 2026 Oct 23;9:1342. doi: 10.1038/s42003-026-11092-0 (PMC13694708; doi:10.1038/s42003-026-11092-0)

# Supplementary Information for

## Dysregulation of the Hippo pathway enhances PD-L2 transcription to promote immune evasion in Head and Neck Squamous Cell Carcinoma

## Supplementary Materials and Methods

### Antibodies and reagents

Anti-BRD4 (67374-2-Ig) was purchased from Proteintech (IL, USA).

### In situ proximity ligation assay (PLA)

In situ PLA was performed as described previously[59].

### DNA constructs

The plasmid HA-TAZ (Addgene 32839)[58] was used.

# Supplementary Figures

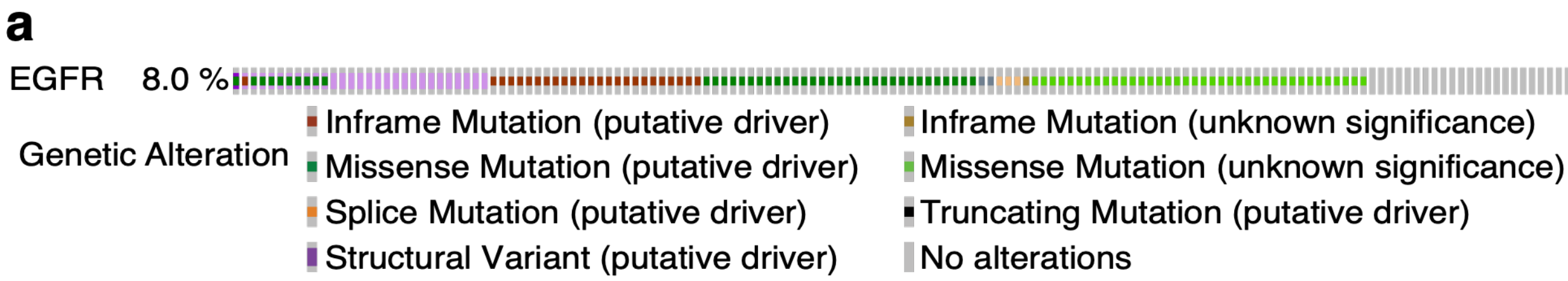


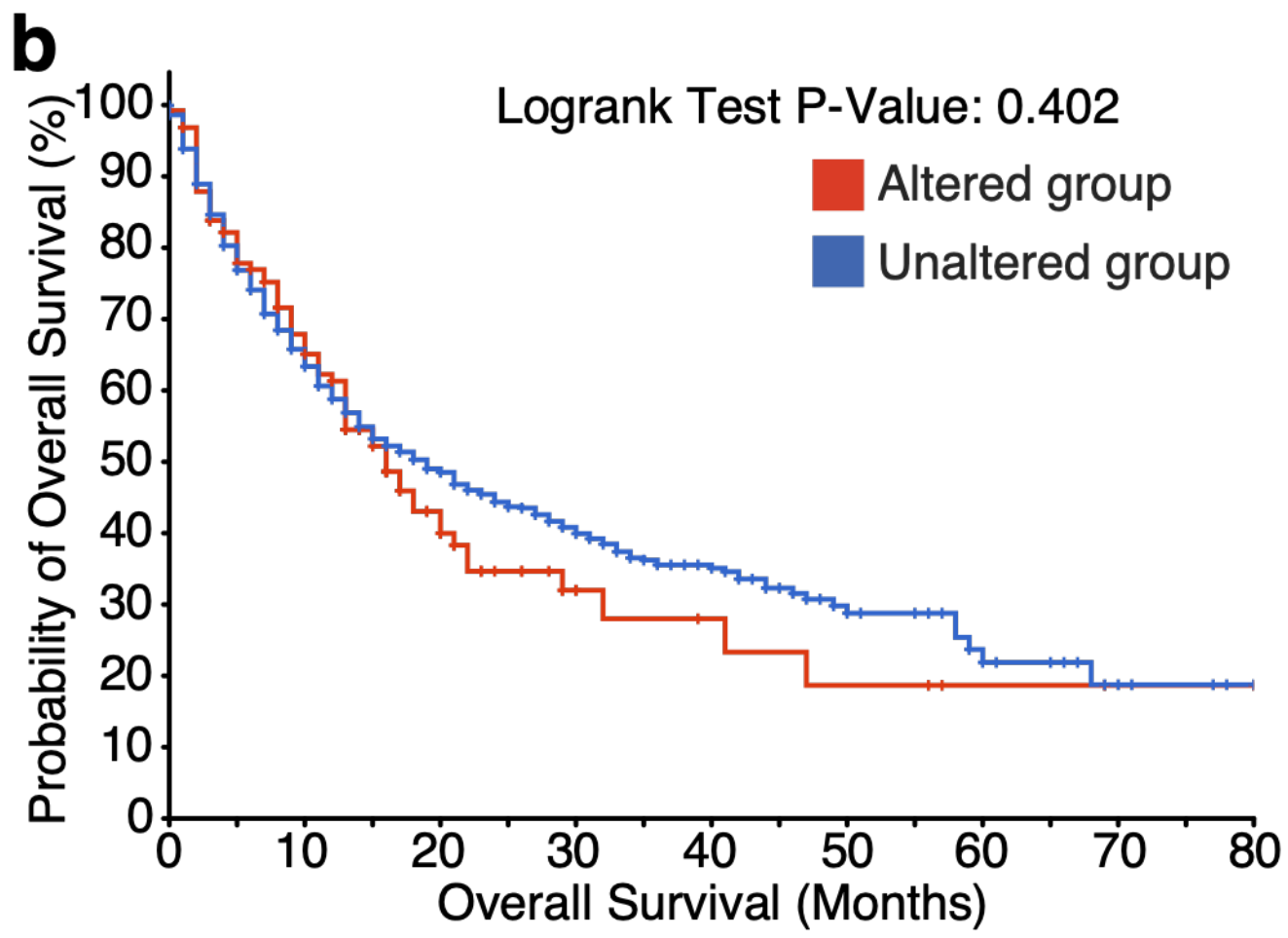


**c**

**Mutation Count and TMB**

| Median (Log2) | Unaltered | Altered | p-Value |
|---|---|---|---|
| Mutation Count | 2.58 | 2.32 | 0.754 |
| TMB | 2.55 | 2.33 | 0.611 |

**Supplementary Figure 1**

**Supplementary Figure 1: EGFR mutations fail to sensitize cancer cells to immune checkpoint inhibitors**

(**a**) Frequency of gene alterations of *EGFR*. (**b**) Overall survival of altered and unaltered groups. The altered group included patients with cancers harboring *EGFR* mutation*s.* (**c**) Mutation counts and tumor mutational burden (TMB) in the altered and unaltered groups. The Student's t-test was used (**c**).

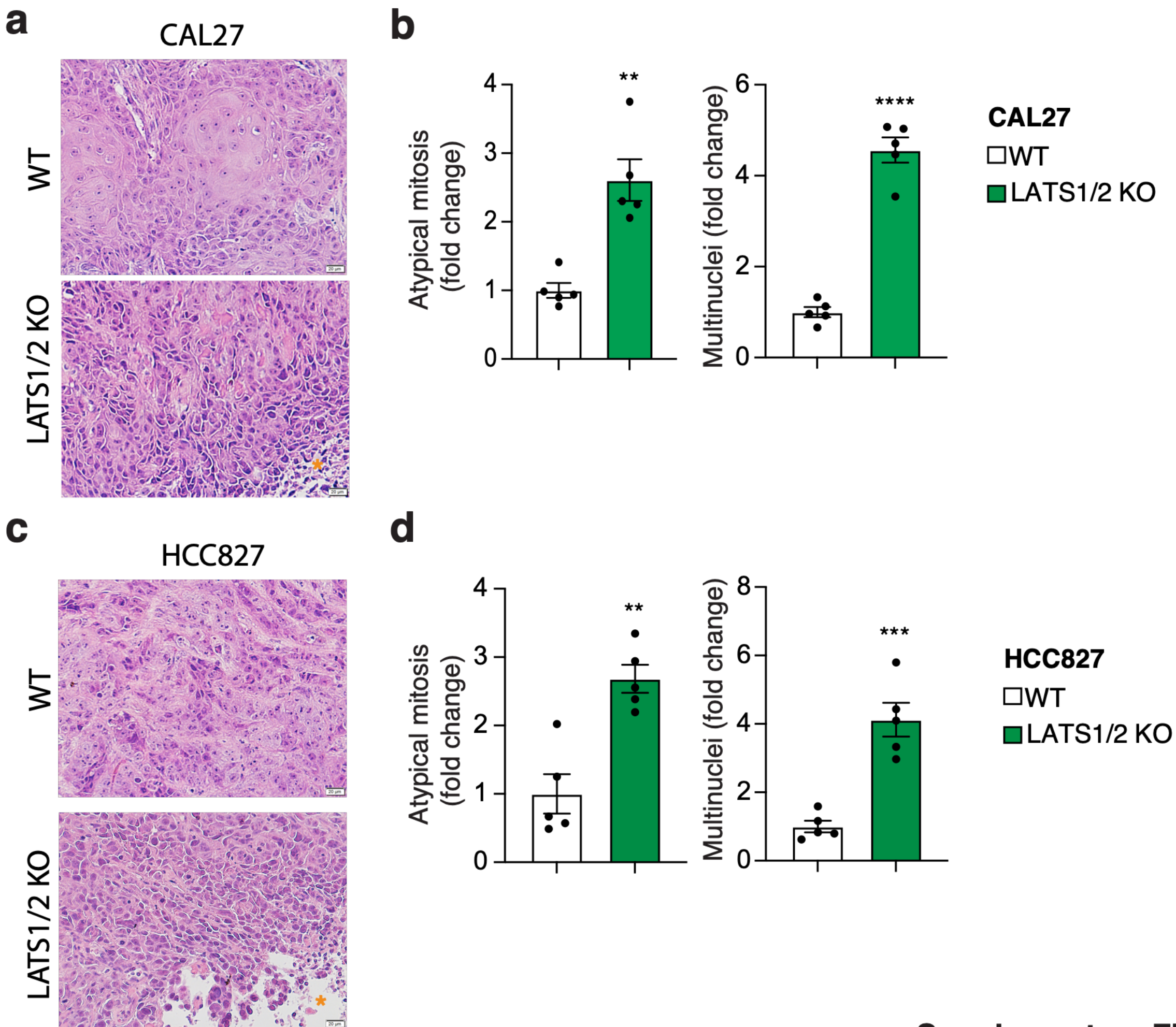


**Supplementary Figure 2**

**Supplementary Figure 2: YAP/TAZ activation is associated with loss of squamous differentiation and cellular atypia**

(**a**) WT and LATS1/2 KO CAL27 cells were injected into the flank of male nude mice, resulting in tumor formation, which were analyzed via HE staining. (**b**) The graphs depict atypical mitosis and multinuclei in the tissue section. Orange asterisk indicates the necrotic area. (**c, d**) HCC827 cells. The Student's t-test was used. Mean±SEM (**b** and **d**); **** $P<0.0001$; *** $P<0.001$; ** $P<0.01$; * $P<0.05$.

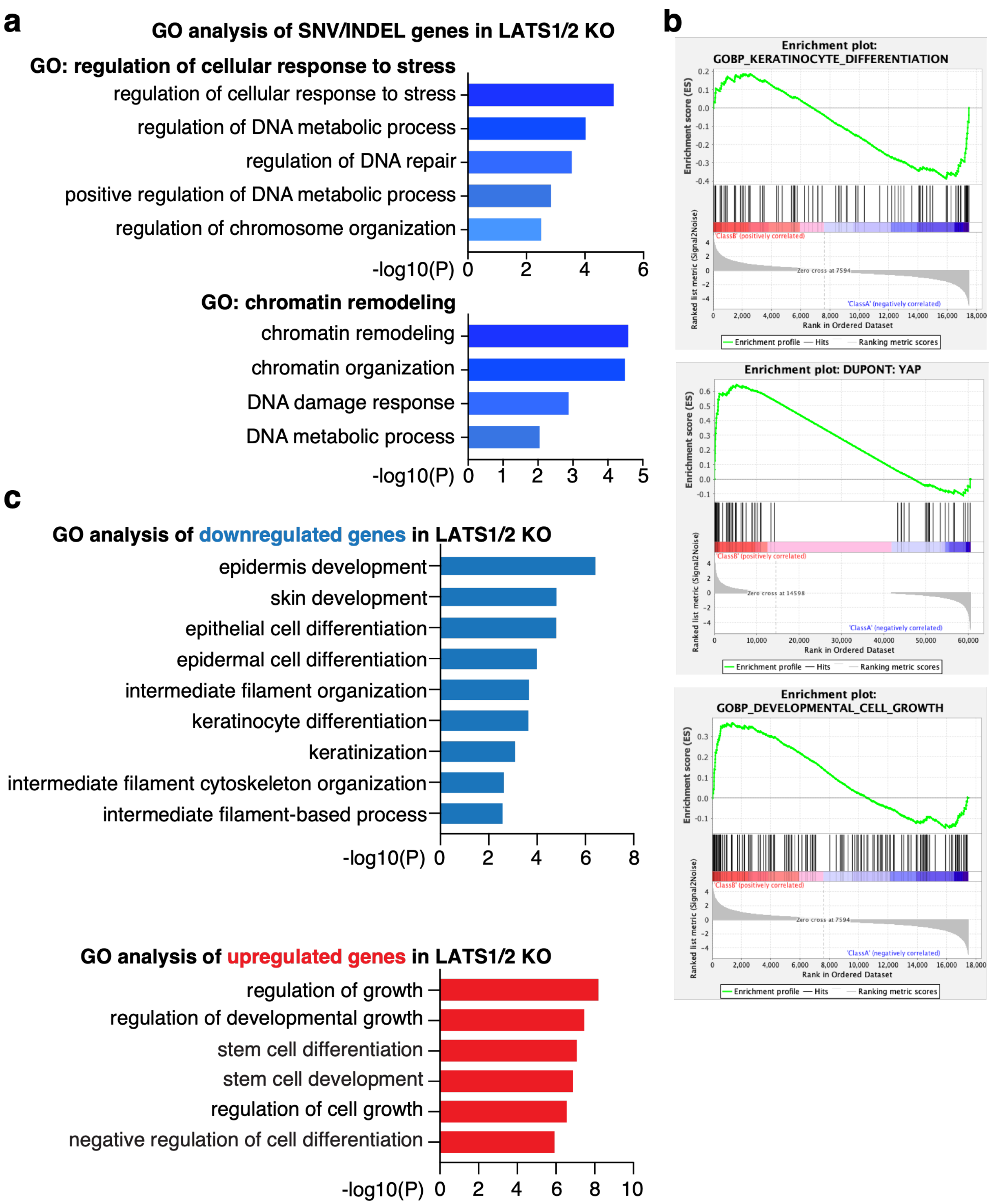


**Supplementary Figure 3**

**Supplementary Figure 3: YAP/TAZ activation reduces epidermal differentiation genes and increases mutations in DNA repair or chromatin remodeling genes**

(**a**) GO analysis of SNVs/INDELs in LATS1/2 KO cells compared to WT CAL27 cells. (**b**) GSEA result of WT vs. LATS1/2 KO CAL27 cells. (**c**) GO analysis of downregulated and upregulated genes in LATS1/2 KO cells compared with those in WT CAL27 cells.

**a**

**Enrichment analysis of YAP signatures (CORDENONSI_YAP_CONSERVED_SIGNATURE) in TCGA, PanCancer Atlas (N=10960) (Only genes with P<0.05, FDR<0.25 are shown)**

| Rank | Gene | NES | NOM p-val | FDR q-val |
|---|---|---|---|---|
| 1 | CMKLR1 | 2.16 | 0.0 | 0.002 |
| 2 | PDCD1LG2 | 2.05 | 0.0 | 0.01 |
| 3 | HLA-DQA1 | 2.01 | 0.0 | 0.0024 |
| 3 | HLA-E | 2.00 | 0.0 | 0.0021 |
| 5 | HLA-DRB1 | 1.95 | 0.004 | 0.0035 |
| 6 | CD274 | 1.67 | 0.025 | 0.067 |
| 7 | CD276 | 1.63 | 0.034 | 0.095 |

**Enrichment analysis of YAP signatures (CORDENONSI_YAP_CONSERVED_SIGNATURE) in Cancer Cell Line Encyclopedia, 2019 (N=1739) (Only genes with P<0.05, FDR<0.25 are shown)**

| Rank | Gene | NES | NOM p-val | FDR q-val |
|---|---|---|---|---|
| 1 | CD276 | 2.07 | 0.0 | 0.0008 |
| 2 | PDCD1LG2 | 1.93 | 0.0 | 0.0046 |
| 3 | STAT1 | 1.85 | 0.002 | 0.0052 |
| 4 | CMKLR1 | 1.63 | 0.029 | 0.054 |

**Supplementary Figure 4**

**Supplementary Figure 4: YAP/TAZ activity is positively correlated with PD-L2**

(**a**) GSEA results of T cell-inflamed GEPs in TCGA, PanCancer Atlas, and Cancer Cell Line Encyclopedia, 2019. Among T cell-inflamed GEPs, only the genes showing $P<0.05$ and $FDR<0.25$ are shown.

**a**

**Enrichment analysis of *PDCD1LG2* in TCGA, PanCancer Atlas (N=10960)**

| Rank | Gene | NES | NOM p-val | FDR q-val |
|---|---|---|---|---|
| 1 | BMI1_DN_MEL18_DN.V1_UP | 2.42 | 0.0 | 0.017 |
| 2 | SNF5_DN.V1_UP | 2.39 | 0.0 | 0.0095 |
| 3 | RPS14_DN.V1_UP | 2.37 | 0.0 | 0.0063 |
| 4 | MEL18_DN.V1_UP | 2.35 | 0.0 | 0.0053 |
| 5 | BMI1_DN.V1_UP | 2.30 | 0.0 | 0.0064 |
| 6 | KRAS.LUNG_UP.V1_UP | 2.17 | 0.0 | 0.0076 |
| 7 | EGFR_UP.V1_UP | 2.10 | 0.0 | 0.0093 |
| 8 | HOXA9_DN.V1_UP | 2.10 | 0.0 | 0.0084 |
| 9 | CORDENONSI_YAP_CONSERVED_SIGNATURE | 2.05 | 0.0 | 0.010 |
| 10 | DUPONT: YAP | 2.03 | 0.0 | 0.011 |
| 11 | LTE2_UP.V1_DN | 2.01 | 0.0 | 0.012 |
| 12 | ZHAO: INDUCED BY YAP | 1.98 | 0.0 | 0.014 |

**Enrichment analysis of *PDCD1LG2* in Cancer Cell Line Encyclopedia, 2019 (N=1739)**

| Rank | Gene | NES | NOM p-val | FDR q-val |
|---|---|---|---|---|
| 1 | BMI1_DN_MEL18_DN.V1_UP | 2.38 | 0.0 | 0.0 |
| 2 | BMI1_DN.V1_UP | 2.29 | 0.0 | 0.0 |
| 3 | MEL18_DN.V1_UP | 2.26 | 0.0 | 0.0 |
| 4 | ESC_V6.5_UP_EARLY.V1_DN | 2.19 | 0.0 | 0.0 |
| 5 | CAHOY_ASTROGLIAL | 2.18 | 0.0 | 0.0 |
| 6 | ZHANG: TEAD-YAP/TAZ | 2.08 | 0.0 | 0.0 |
| 7 | EGFR_UP.V1_UP | 2.05 | 0.0 | 0.0 |
| 8 | PTEN_DN.V2_UP | 2.02 | 0.0 | 0.0 |
| 9 | LEF1_UP.V1_UP | 1.98 | 0.0 | 0.0 |
| 10 | STK33_NOMO_UP | 1.98 | 0.0 | 0.0 |
| 11 | DUPONT: YAP | 1.96 | 0.0 | 0.0 |
| 12 | CORDENONSI_YAP_CONSERVED_SIGNATURE | 1.93 | 0.0 | 0.0 |

**b**

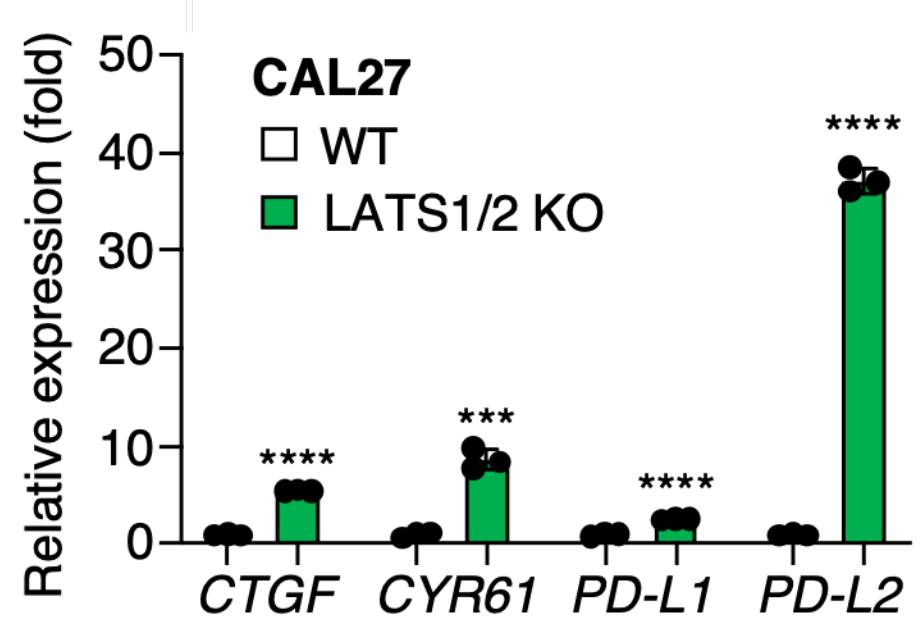


**Supplementary Figure 5**

**Supplementary Figure 5: YAP/TAZ predominantly promotes PD-L2 expression**

(**a**) GSEA results of *PDCD1LG2* in TCGA, PanCancer Atlas, and Cancer Cell Line Encyclopedia. YAP-regulated signature gene sets are highlighted in red. (**b**) GO analysis of SNVs/INDELs in LATS1/2 KO cells compared to WT CAL27 cells. Relative mRNA expression levels of *CTGF*, *CYR61*, *PD-L1*, and *PD-L2* in WT and LATS1/2 KO CAL27 cells. The Student's t-test was used. Mean±SEM (**b**); **** $P$<0.0001; *** $P$<0.001.

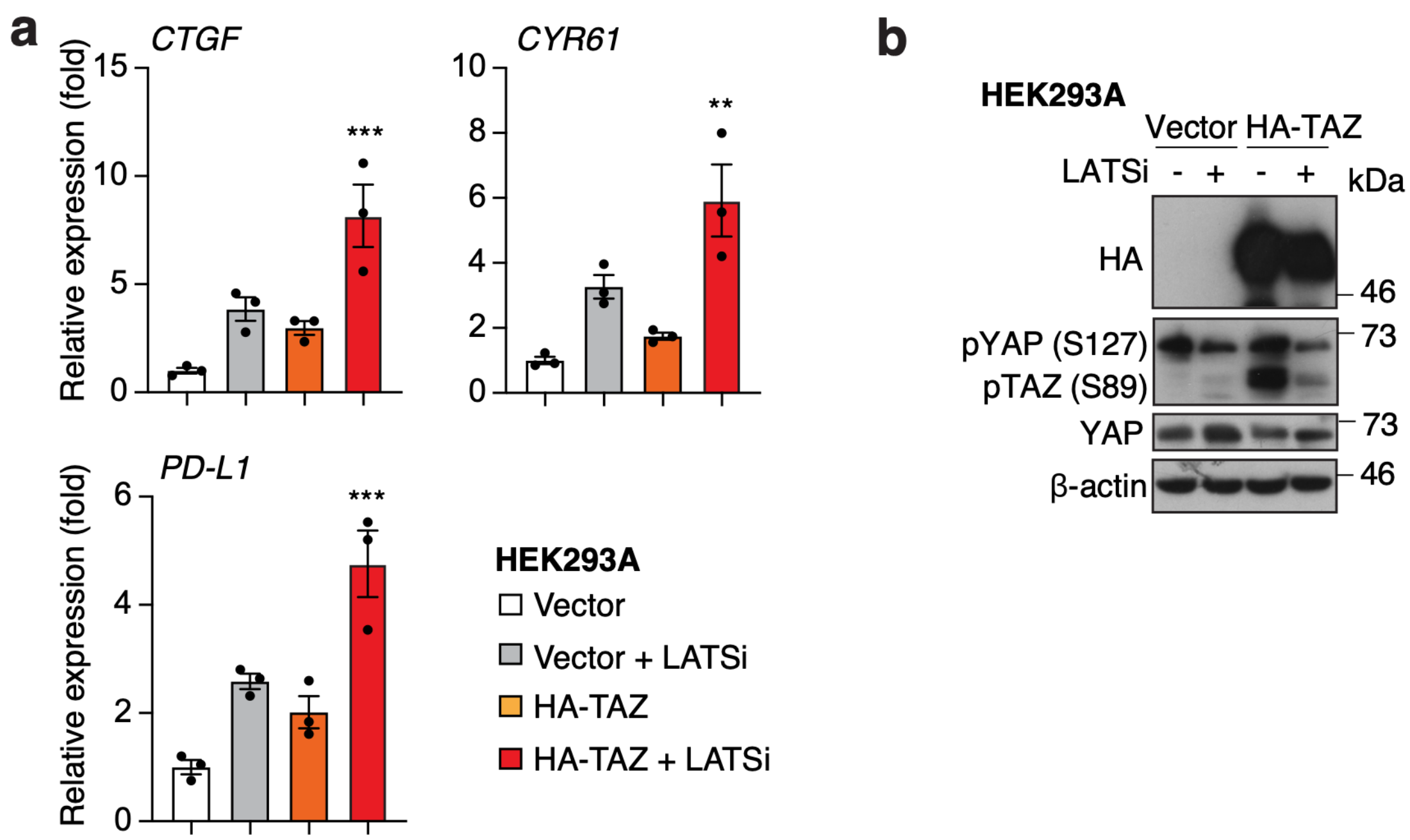


Supplementary Figure 6

**Supplementary Figure 6: TAZ predominantly promotes *PD-L1* expression, not *PD-L2***

(**a**) Relative mRNA expression of *CTGF*, *CYR61*, and *PD-L1* in HEK293A cells overexpressing vector or HA-TAZ. The cells were treated with LATS inhibitor (LATS-IN-1) at 10 μM for 24 h. (**b**) Immunoblot of HA, pYAP (S127), pTAZ (S89), YAP, and β-actin. ANOVA with the

Tukey−Kramer post hoc test was used. Mean±SEM (**a**); *** $P<0.001$; ** $P<0.01$; *versus Vector (**a**).

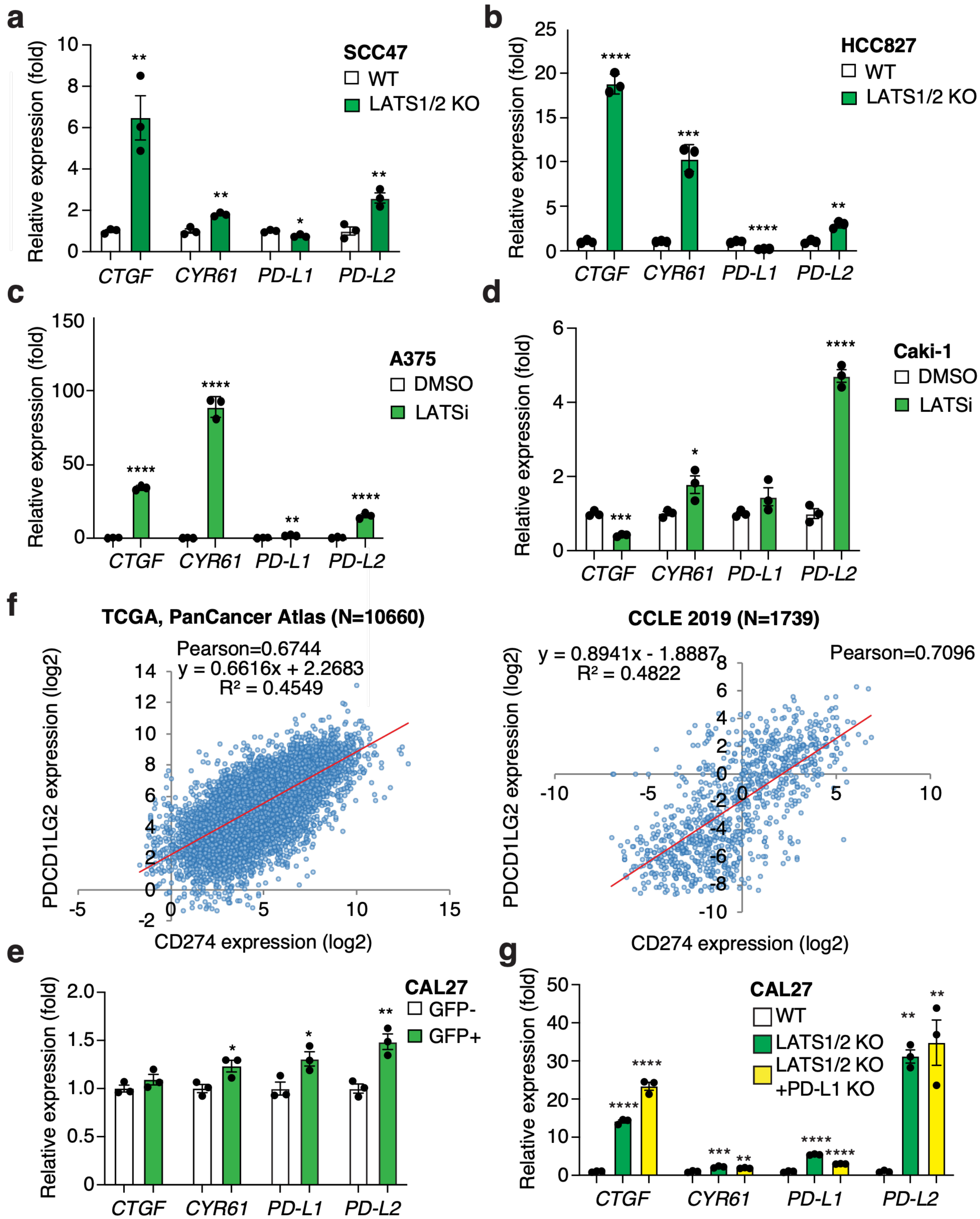


Supplementary Figure 7

**Supplementary Figure 7: YAP/TAZ enhance transcription of *PD-L1* and *PD-L2* in various types of cancers**

(**a**) Relative mRNA expression of *CTGF*, *CYR61*, *PD-L1*, and *PD-L2* in WT and LATS1/2 KO SCC47 cells. (**b**) HCC827 cells. (**c**) Relative mRNA expression of *CTGF*, *CYR61*, *PD-L1*, and *PD-L2* in A375 cells. The cells were then serum starved with treatment of LATS inhibitor (LATS-IN-1) at 10 μM for 24 h. (**d**) Caki-1 cells. (**e**) Relative mRNA expression of *CTGF*, *CYR61*, *PD-L1*, and *PD-L2* of GFP-inducible CAL27 cells. Cells were treated with DMSO or doxycycline for 48 h. (**f**) Graphs showing the correlation between *CD274* (*PD-L1*) and *PDCD1LG2* (*PD-L2*) in TCGA, PanCancer Atlas, and CCLE 2019. (**g**) Relative mRNA expression levels of *CTGF*, *CYR61*, *PD-L1*, and *PD-L2* in WT, LATS1/2 KO, and LATS1/2 KO + PD-L1 KO CAL27 cells. ANOVA with the Tukey−Kramer post hoc test was used. The Student's t-test was used. Mean±SEM (**a**-**e**, and **g**); **** $P<0.0001$; *** $P<0.001$; ** $P<0.01$; * $P<0.05$; *versus WT (**g**).

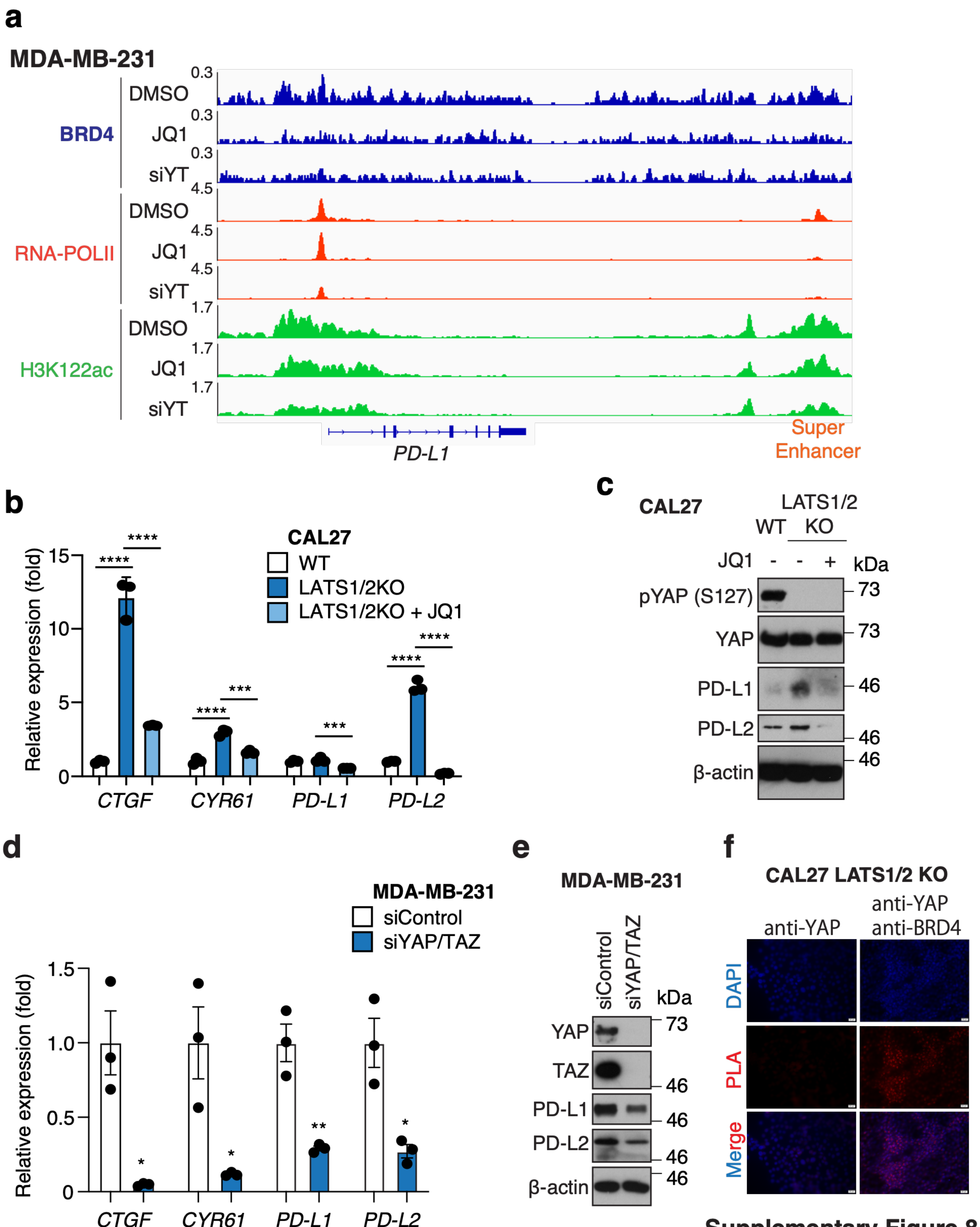


Supplementary Figure 8

**Supplementary Figure 8: YAP/TAZ/TEAD, BRD4, and POLII are located on the super-enhancer and promoter, enhancing *PD-L1* and *PD-L2* transcription**

(**a**) ChIP-seq results for BRD4, RNA-POLII, and H3K122ac expression in MDA-MB-231 cells. Cells were treated with DMSO, a BRD4 inhibitor (JQ1), or transfected with siRNA for YAP/TAZ. (**b**) Relative mRNA expression levels of *CTGF*, *CYR61*, *PD-L1*, and *PD-L2* in WT, LATS1/2 KO, and LATS1/2 KO + JQ1-treated cells. Cells were treated with DMSO or JQ1 (1 μM) for 24 h. (**c**) Immunoblot of pYAP (S127), YAP, PD-L1, PD-L2, and β-actin. (**d**) Relative mRNA expression levels of *CTGF*, *CYR61*, *PD-L1*, and *PD-L2* in MDA-MB-231 cells transfected with siRNA for negative control or YAP/TAZ. (**e**) Immunoblot of YAP, TAZ, PD-L1, PD-L2, and β-actin. (**f**) Immunofluorescence of proximity ligation assay in LATS1/2 KO CAL27 cells. The red signal suggests the protein interaction. The antibodies against YAP and BRD4 were used. ANOVA with Tukey−Kramer post hoc test was used. The Student's t-test was used. Mean±SEM (**b** and **d**); **** $P<0.0001$; *** $P<0.001$; ** $P<0.01$; * $P<0.05$. *versus LATS1/2 KO (**b**).

Fig 3d

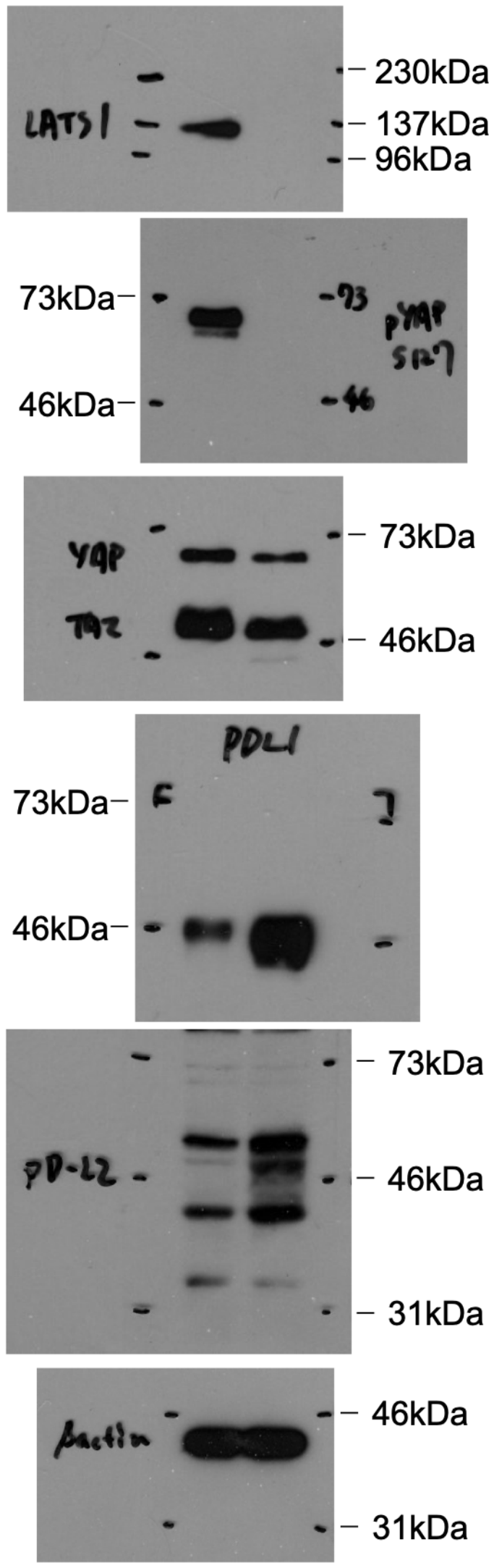

230kDa
LATS1
137kDa
96kDa
73kDa
pYAP S127
46kDa
YAP
73kDa
TAZ
46kDa
PDL1
73kDa
46kDa
73kDa
PD-L2
46kDa
31kDa
βactin
46kDa
31kDa


Fig 3e

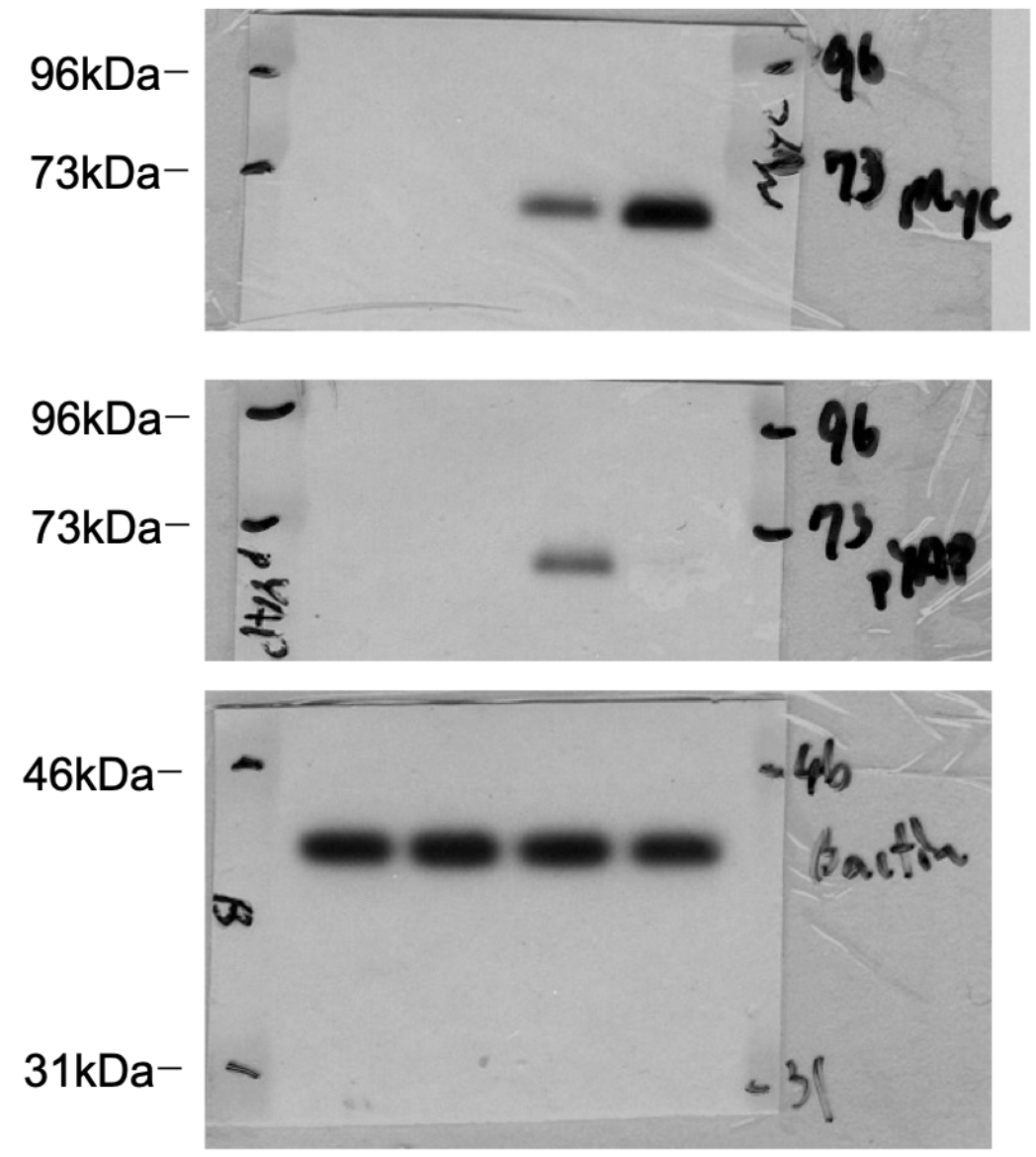

96kDa
73kDa
pMyc
96kDa
73kDa
pYAP
46kDa
βactin
31kDa

Fig 4c

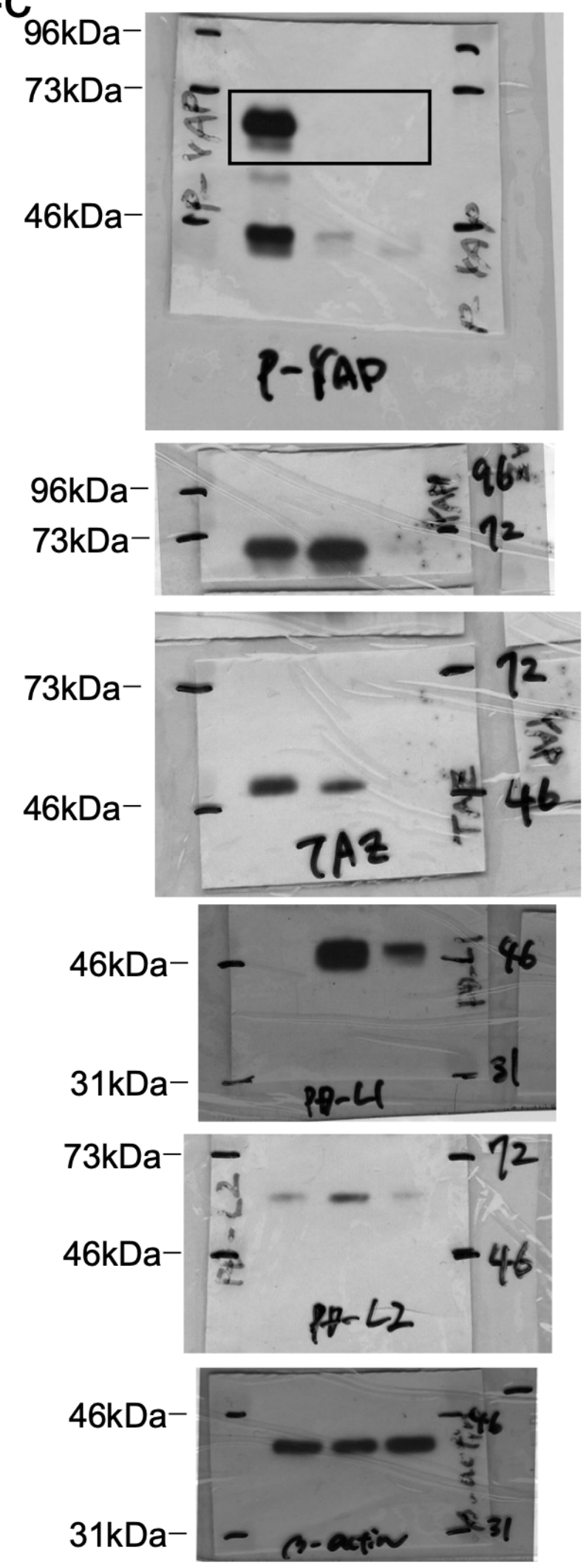


Fig 4d

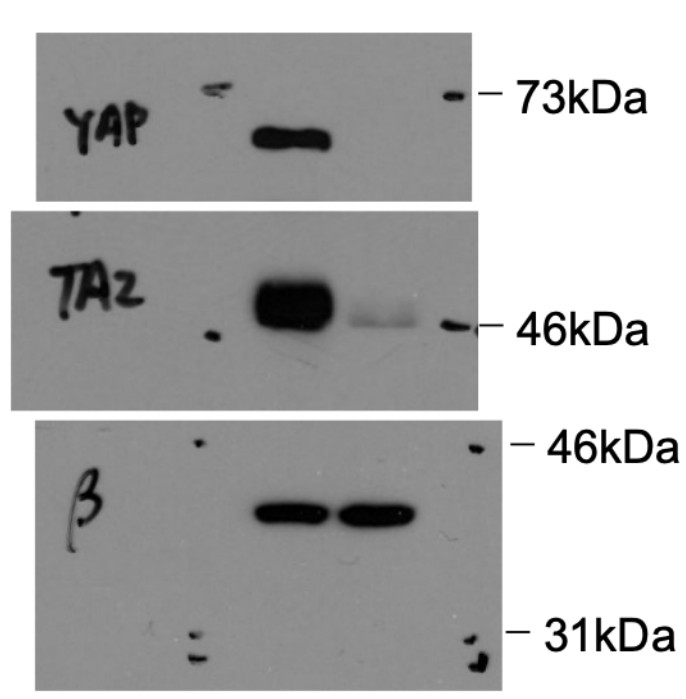


Fig 4g

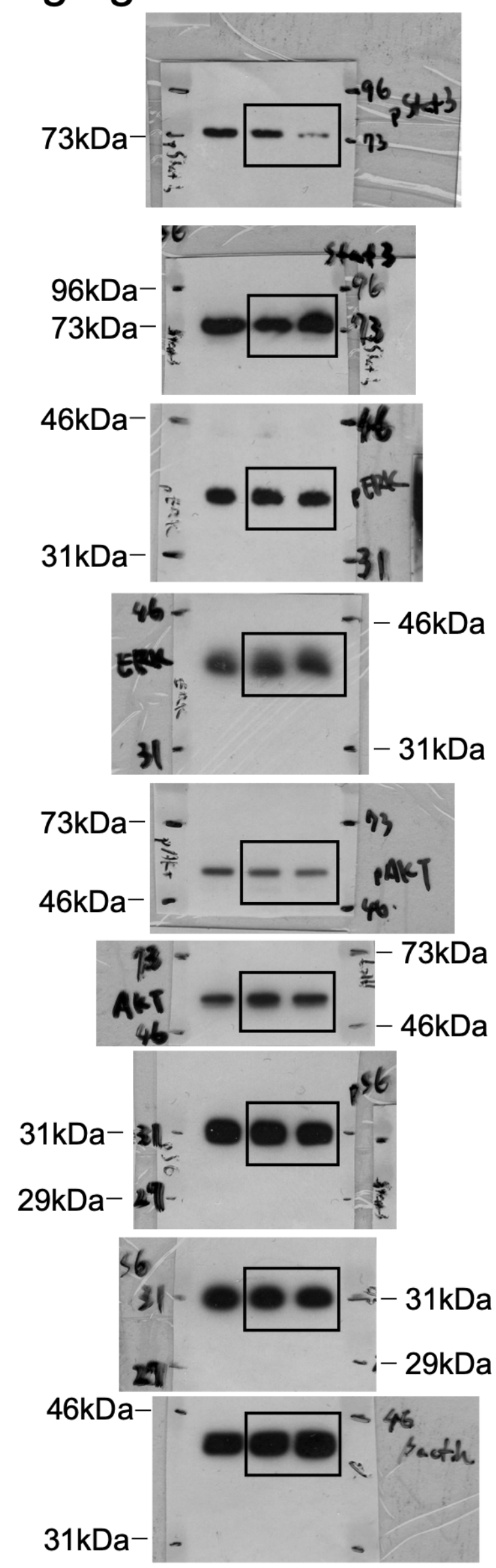

Supplementary Fig 6b

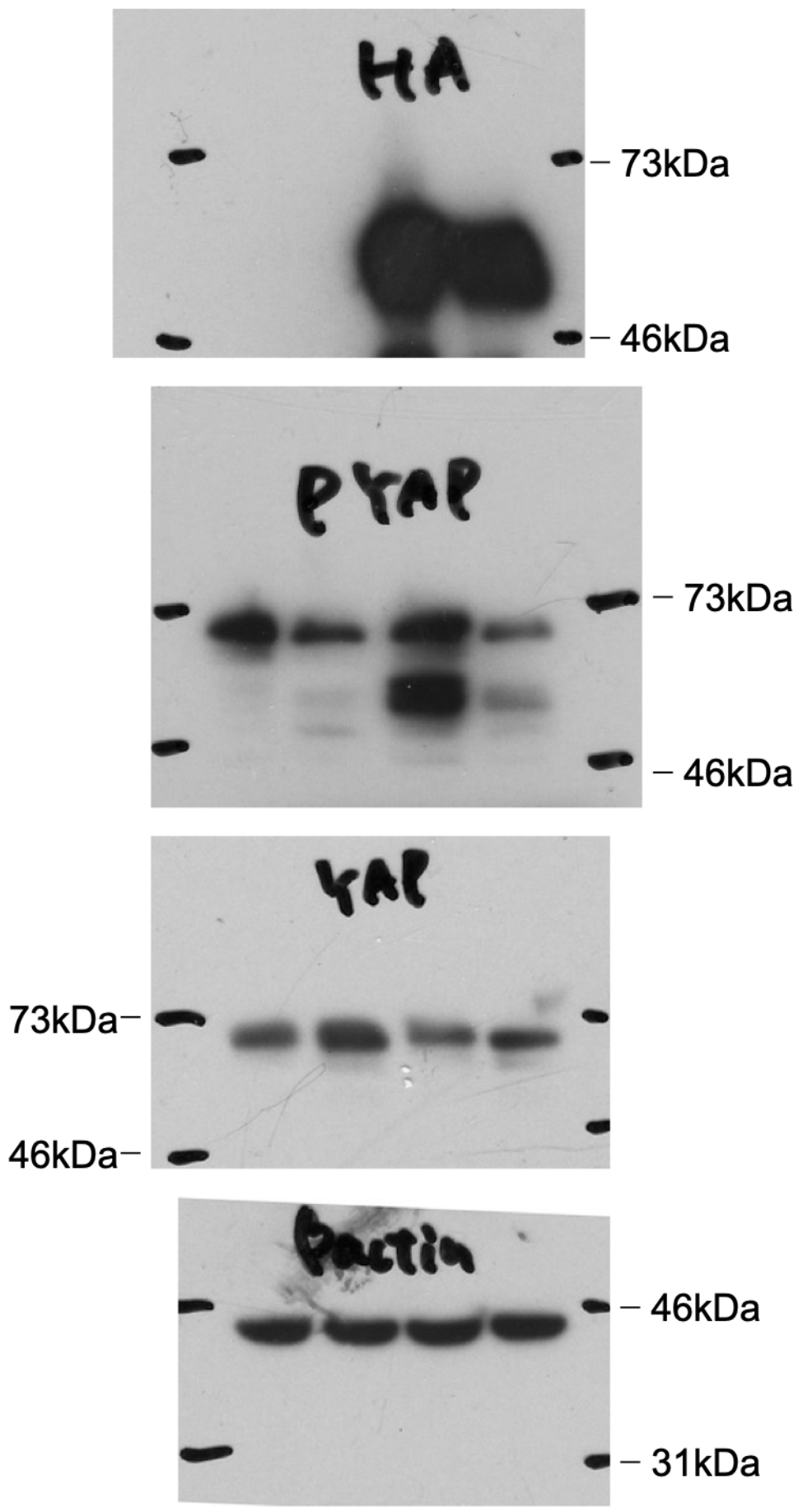

Supplementary Fig 8c

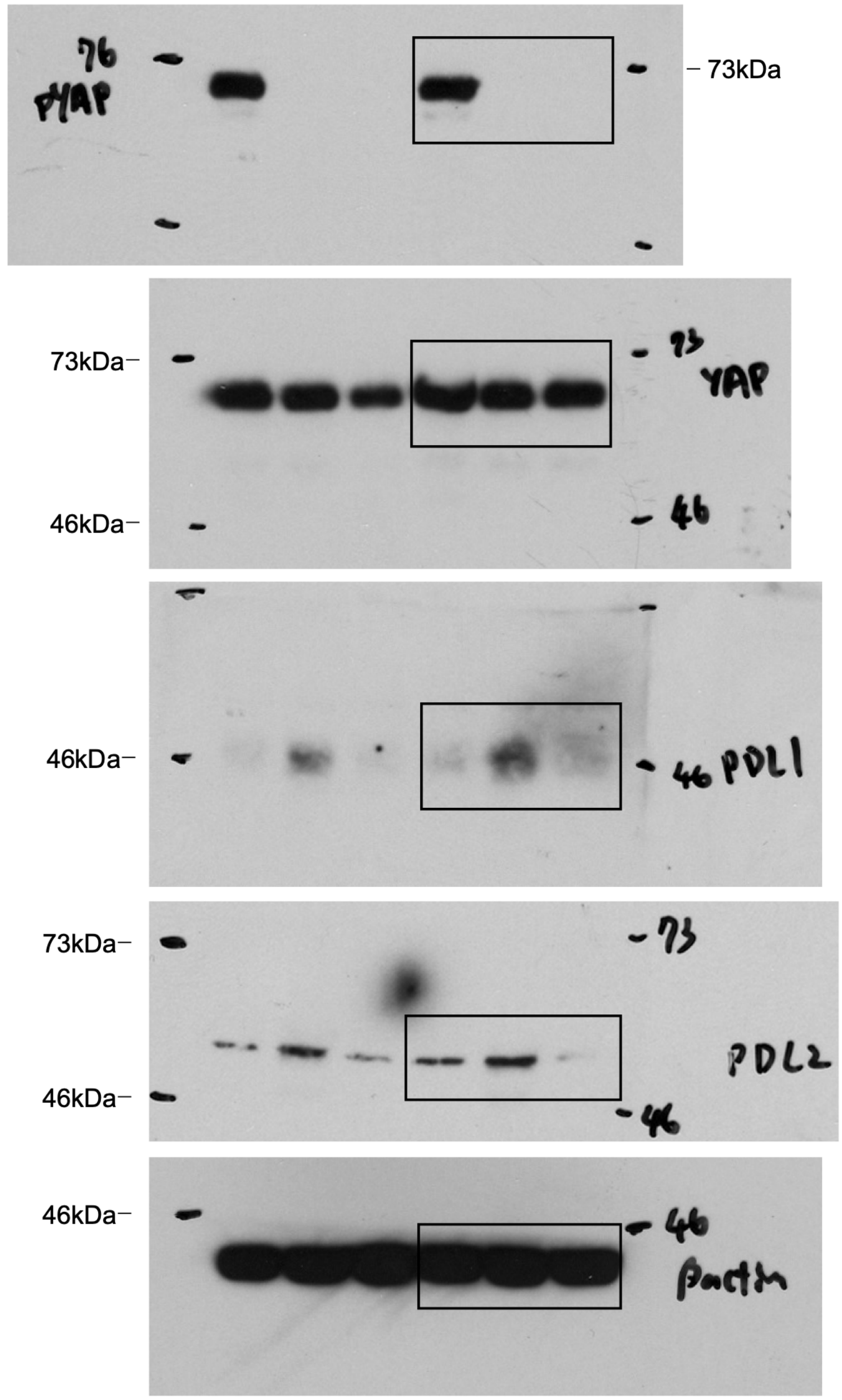

Supplementary Fig 8e

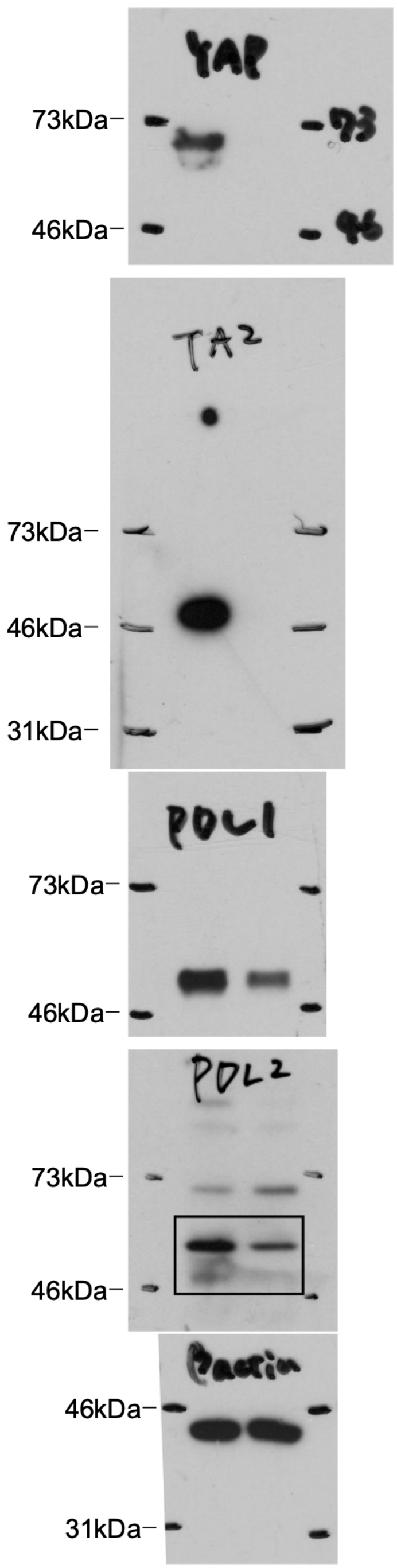

Supplement: Supplementary file 1 [file 42003_2026_11092_MOESM1_ESM.pdf]
