# Supplementary material for: Dysregulation of the Hippo pathway enhances PD-L2 transcription to promote immune evasion in head and neck squamous cell carcinoma
Source: Commun Biol. 2026 Oct 23;9:1342. doi: 10.1038/s42003-026-11092-0 (PMC13694708; doi:10.1038/s42003-026-11092-0)
Supplement: Supplementary file 2 [file 42003_2026_11092_MOESM2_ESM.pdf]

Description of Additional Supplementary Files

Supplementary Data 1- Source data for the figures in this paper are provided in Supplementary Data 1
